# Characterization of H/D exchange in type 1 pili by proton-detected solid-state NMR and molecular dynamics simulations

**DOI:** 10.1007/s10858-019-00247-3

**Published:** 2019-04-26

**Authors:** Songhwan Hwang, Carl Öster, Veniamin Chevelkov, Karin Giller, Sascha Lange, Stefan Becker, Adam Lange

**Affiliations:** 1 Department of Molecular Biophysics, Leibniz-Forschungsinstitut für Molekulare Pharmakologie (FMP), Berlin, Germany; 2 Department of NMR-Based Structural Biology, Max Planck Institute for Biophysical Chemistry, Göttingen, Germany; 3 Institut für Biologie, Humboldt-Universität Zu Berlin, Berlin, Germany

**Keywords:** Uropathogenic *E*. *coli*, Type 1 pili, Hydrogen deuterium exchange, Solid-state NMR, Molecular dynamics simulations

## Abstract

Uropathogenic *Escherichia coli* invades and colonizes hosts by attaching to cells using adhesive pili on the bacterial surface. Although many biophysical techniques have been used to study the structure and mechanical properties of pili, many important details are still unknown. Here we use proton-detected solid-state NMR experiments to investigate solvent accessibility and structural dynamics. Deuterium back-exchange at labile sites of the perdeuterated, fully proton back-exchanged pili was conducted to investigate hydrogen/deuterium (H/D) exchange patterns of backbone amide protons in pre-assembled pili. We found distinct H/D exchange patterns in lateral and axial intermolecular interfaces in pili. Amide protons protected from H/D exchange in pili are mainly located in the core region of the monomeric subunit and in the lateral intermolecular interface, whereas the axial intermolecular interface and the exterior region of pili are highly exposed to H/D exchange. Additionally, we performed molecular dynamics simulations of the type 1 pilus rod and estimated the probability of H/D exchange based on hydrogen bond dynamics. The comparison of the experimental observables and simulation data provides insights into stability and mechanical properties of pili.

**Electronic supplementary material:**

The online version of this article (10.1007/s10858-019-00247-3) contains supplementary material, which is available to authorized users.

## Introduction

**Fig. 1:**
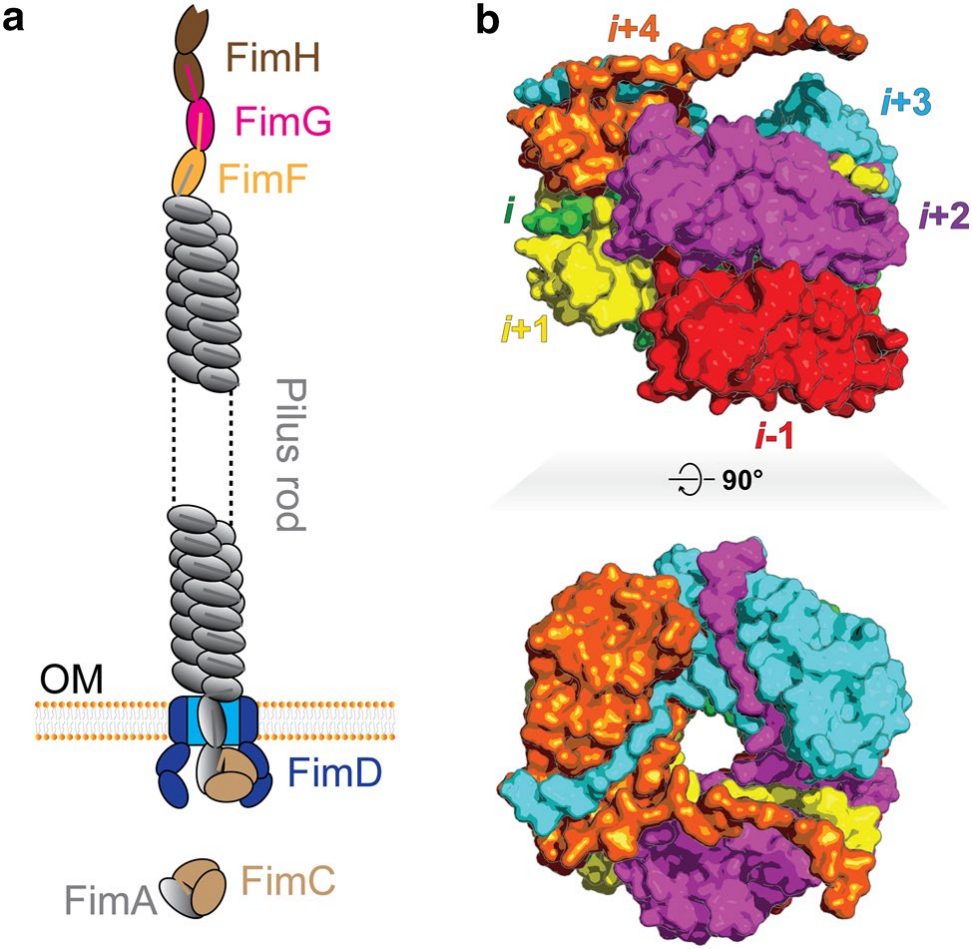
Structural composition of the type 1 pilus. **a** Schematic diagram of the type 1 pilus including the tip (FimH, FimG, and FimF), the pilus rod (FimA), the usher (FimD), and the chaperone (FimC). The pilus is embedded in the outer membrane (OM). (b) Side view (upper panel) and top view (lower panel) of the structure of the type 1 pilus rod (PDB entry 5OH0) (Hospenthal et al. 2017)

Uropathogenic *Escherichia coli* (UPEC) is the primary pathogen causing urinary tract infections by adhesion to the surface of host bladder epithelial cells (Martinez et al. 2000). The adhesion is a prerequisite for colonization and invasion of host cells, and consequently, pili constitute an important virulence factor (Mulvey et al. 1998). The extracellular component of the type 1 pilus is composed of a short tip fibrilium, made up of a two-domain adhesin (FimH) and two adaptors (FimG and FimF), and a long pilus rod (Fig. 1), consisting of an assembly of thousands of FimA subunits, where the monomeric FimA is a 159 amino acid protein (Waksman and Hultgren 2009). The FimH adhesin recognizes and attaches to mannosylated proteins on the surface of the host cells (Krogfelt et al. 1990). Type 1 pili are highly extensible and can be extended several times the contracted length (~1 μm) from the surface of bacteria without any dissociation of the polymers in a fully reversible process (Miller et al. 2006).

The pilus rod subunit FimA has an incomplete immunoglobulin-like β-sheet fold (pilin body) and is complemented by a protruding N-terminal extension (Nte, termed donor strand) of a neighboring subunit with non-covalent interactions (Choudhury et al. 1999). This donor strand complementation (DSC) is processed by the highly conserved chaperone-usher pathway in the periplasmic space of UPEC with assistance of the chaperone FimC and the usher FimD (Phan et al. 2011; Geibel et al. 2013). Sequential DSCs along pili give rise to a right-handed helical structure (Hahn et al. 2002). While the essential elastic properties of pili originate from their coil-like arrangement of subunits, the stability of pili mainly depends on complementation of conserved P1–P5 residues (Gly8, Val10, Phe12, Gly14, and Val16) in the donor strand (Puorger et al. 2008). Extensibility and stability of pili have presumably evolved to withstand high shear force, preventing the bacteria from being washed away by the flow of bulk urine (Spaulding, C.N. et al. 2018).

The uncoiling process of the pilus rod is typically divided into three distinct regions (I, II, and III) in force versus elongation response. This process has been investigated by force spectroscopic techniques including optical tweezers (Andersson et al. 2008) and atomic force microscopy (Forero et al. 2006). In region I, the entire pilus rod is stretched without significant conformational change. In region II, uncoiling of the pilus rod at a constant force is a consequence of breakages in layer-to-layer interactions (e.g. subunit *i* and subunit *i*+3) along the pilus rod. In region III, the pilus rod undergoes conformational changes in its head-to-tail region (e.g. subunit *i*−1 and subunit *i*) while the pilus rod is completely stretched out under higher forces than the constant force in region II. The uncoiled pilus rod can be spontaneously coiled and the restoring force can also be divided into the three stages (I, II, and III). These reversible uncoiling and coiling properties of the pilus rod enable UPEC to resist shear force from bulk urine flow.

For structural studies of inherently insoluble and non-crystalline pili, solid-state NMR and cryo-electron microscopy (cryo-EM) are advantageous methods. We have previously determined a high-resolution model of type 1 pili by combining ^13^C-detected solid-state NMR, scanning transmission electron microscopy, and inferential structure determination (Habenstein et al. 2015). The protomer in the pilus model was estimated from the self-complemented monomeric FimA structure, which was determined by solution NMR (PDB entry 2JTY) (Puorger et al. 2011). Recently, cryo-EM structures of type 1 pili were determined at a resolution of 4.2 Å (Spaulding et al. 2018; Hospenthal et al. 2017). The structure of the other major type of pili in UPEC (P pili) was determined by cryo-EM at a resolution of 3.8 Å (Hospenthal et al. 2016). A comparison between the solid-state NMR structure and the cryo-EM structures of type 1 pili reveals that their quaternary structures are different while monomeric structures of the pilin body and the donor strand are comparable. The solid-state NMR structure exhibited a highly coiled state with a shorter axial rise per subunit and a higher number of subunits per turn compared to the cryo-EM structures. This difference might originate from the different assembly pathways (in vitro for solid-state NMR versus in vivo for cryo-EM) as described by Hospenthal et al. (2017).

Here we use proton-detected fast magic-angle spinning (MAS) solid-state NMR spectroscopy of perdeuterated, fully proton back-exchanged pili to observe ^1^H^N^ (amide proton), ^13^C, and ^15^N backbone resonances. Fast MAS of perdeuterated proteins with subsequent proton back-exchange has become a valuable method to study structure and dynamics of proteins by proton-detected solid-state NMR (Chevelkov et al. 2006; Lamley et al. 2014; Fricke et al. 2017; Najbauer et al. 2019). Proton-detection requires less amount of sample and provides higher sensitivity compared to ^13^C/^15^N-detection due to the higher gyromagnetic ratio of ^1^H. We achieved nearly complete backbone chemical shift assignments by conducting multi-dimensional solid-state NMR experiments, where assignments are missing for three residues at the N-terminus. We also investigated the degree of hydrogen/deuterium (H/D) exchange at labile sites in perdeuterated and fully proton back-exchanged pili. The previous non-equilibrium H/D exchange method by solution NMR (Wagner and Wüthrich 1982) was adapted for solid-state NMR: the degree of H/D exchange in a protonated protein in D_2_O buffer is measured after a certain incubation time (Gallagher et al. 1992; Whittemore et al. 2005; Wang et al. 2011; Medeiros-Silva et al. 2017; Grohe et al. 2017; Medeiros-Silva et al. 2016). A similar strategy has been applied to investigate the type iii secretion system needle protein (Chevelkov et al. 2017). The H/D exchange rate of amide protons depends on formation and breakage of hydrogen bonds, local conformational distortion, neighboring side-chains, and accessibility of water and hydroxide ions (OH^-^) to labile sites (Bai et al. 1993; Englander et al. 1996). Therefore, it is interesting to compare the extent of H/D exchange with the dynamic properties of pili in liquid phase (Maity et al. 2003). In addition to the H/D exchange experiments, we performed molecular dynamics (MD) simulations (McAllister and Konermann 2015; Persson and Halle 2015) on type 1 pili. From the simulations we estimated the probability of H/D exchange by counting the number of hydrogen bonds between backbone H-N and water in the absence of hydrogen bonds between the backbone H-N and other residues.

## Materials and methods

### Sample preparation

Uniformly [^2^H,^13^C,^15^N]-labeled recombinant FimA was expressed in *E*. *coli* strain BL21 (DE3) using fully deuterated minimal medium with ^15^N-ammonium chloride and [^2^H,^13^C]-glucose as the sole nitrogen and carbon sources, respectively, as described before (Habenstein et al. 2015). The sample perdeuteration is required to dilute the strong network of proton-proton dipolar couplings that otherwise significantly impair spectral resolution. The perdeuterated pili sample was purified under denaturing conditions and kept at 37 °C for three weeks in 10 mM sodium phosphate buffer at pH 7.0 with 0.02 % sodium azide in H_2_O (non-denaturing 100 % H_2_O buffer) to produce fully proton back-exchanged pili (protonation at all labile sites), herein referred to as reprotonated pili (Fig. S1). The pili sample was then collected by centrifugation at 48,700×*g* and the pellet was packed into a 1.9 mm rotor with addition of a few crystals of DSS (4,4-dimethyl-4-silapentane-1-sulfonic acid) and a small amount of D_2_O (~ 1 μL) for a field lock on the deuterons.

For the preparation of a redeuterated pili sample (Fig. S1), a pre-assembled reprotonated pili sample was washed with non-denaturing 100% D_2_O buffer [10 mM sodium phosphate at pH 7.0, 0.02 % sodium azide in D_2_O; prepared as described in the protocol (Fricke et al. 2017)] and incubated at room temperature for 4 h followed by ultracentrifugation at 48,700×*g*. These washing and pelleting procedures were repeated three more times. This leads to that only solvent accessible labile protons will be exchanged to deuterons in contrast to the initial reprotonation, which was performed before and during assembly of the pili and resulted in complete proton back-exchange of exchangeable sites. The redeuterated pili sample was packed into a 1.9 mm rotor with a few crystals of DSS. The amount of the reprotonated sample and the redeuterated sample in each rotor is approximately equal.

### Solid-state NMR spectroscopy

Proton-detected solid-state NMR experiments were performed on 21.2-T and 16.4-T (^1^H Larmor frequencies of 900 MHz and 700 MHz, respectively) spectrometers (Bruker Biospin, Germany) equipped with four-channel MAS probeheads (^1^H, ^13^C, ^2^H, ^15^N) for a rotor diameter of 1.9 mm. All the experiments were carried out at an MAS frequency of 40 kHz. The proton DSS signal was set to 0 ppm and used as internal chemical shift reference. The internal sample temperature was monitored by the water proton resonance relative to DSS (Böckmann et al. 2009). Two dimensional (2D) (H)NH and three dimensional (3D) 3D (H)CANH, 3D (H)CONH, 3D (H)COCA(N)H, 3D (H)CA(CO)NH, and 3D (H)CO(CA)NH experiments were conducted for the reprotonated pili on the 21.2-T spectrometer at an internal sample temperature of 22±1 °C. 2D (H)NH and 3D (H)CANH spectra for the reprotonated pili and the redeuterated pili were acquired on the 16.4-T spectrometer at an internal sample temperature of 17±1 °C. Reintroduction of hetero- and homo-nuclear dipolar interactions under MAS was achieved by cross-polarization (CP) (Pines et al. 1972), SPECIFIC-CP (Baldus et al. 1998), and dipolar recoupling enhanced by amplitude modulation (DREAM) (Verel et al. 2001). Decoupling schemes including WALTZ-16 (Shaka et al. 1983) and X inverse-X (XiX) (Ernst et al. 2003) were used during the chemical shift evolution periods. Gaussian pulse cascades (Emsley and Bodenhausen 1992) (Q3 in Bruker nomenclature) were applied for band-selective inversion on Cα or CO. The detailed experimental procedures and pulse sequences of the above experiments are mostly explained in a previously published protocol (Fricke et al. 2017) and experimental parameters are provided in Tables S1–S8. TopSpin 3.2 (Bruker Biospin, Germany) and CcpNmr Analysis 2.2.4 (Vranken, W.F. et al. 2005) were used to process the spectra and to assign chemical shifts, respectively. The 3D (H)CANH spectra of the reprotonated pili and redeuterated pili were recorded on the same 16.4-T spectrometer 5 months after the sample packing of the redeuterated pili in a 1.9 mm rotor. For the purpose of consistency, the 3D (H)CANH spectra of the reprotonated pili and the redeuterated pili were acquired in a row. The peak volumes of assigned peaks were used to compare H/D exchange between redeuterated and reprotonated pili.

To compare the different chemical environments in monomeric FimA and assembled FimA, chemical shift perturbations (CSPs) were calculated using the equation

$${\text{CSP }} = \sqrt {\frac{1}{2}\left[ {\delta_{H}^{2} + \left( {\alpha \cdot \delta_{N}^{2} } \right)} \right]} ,$$

where the chemical shift differences of ^1^H^N^ and ^15^N between solid-state NMR (assembled) and solution NMR (self-complemented monomer) are $\delta_{H} = \delta_{H}^{solid - state NMR} - \delta_{H}^{solution NMR}$ and $\delta_{N} = \delta_{N}^{solid - state NMR} - \delta_{N}^{solution NMR}$, respectively (Williamson 2013). Weighting factors α = 0.2 for glycine and α = 0.14 for other amino acids were used (Fig. S2). The weighting factor is used to account for the difference in spectral width between backbone ^1^H and ^15^N. Since the chemical shift difference for ^15^N compared to ^1^H is smaller for glycine compared to other residues a higher weighting factor is used for glycine (Urbaniak et al. 2002).

### All-atom molecular dynamics simulations

The cryo-EM structure of the type 1 pilus rod containing six FimA subunits (PDB entry 5OH0, 4.2-Å resolution) was selected as the starting structure for molecular dynamics (MD) simulations. The missing amino- and carboxyl-terminal residues in the cryo-EM structure (Ala1 and Gln159) were added using PyMOL 1.3 (Schrödinger 2015). Dominant protonation states of all titratable residues at pH 7.0 were assigned. The protein was immersed in 23054 extended simple point charge (SPC/E) water models (Berendsen et al. 1987) in a box, which ensures that each edge of the box is at least 1 nm apart from the protein in the center of the box. In addition to the solvation, 30 sodium ions and 150 mM sodium chloride replaced randomly the same number of water models to neutralize the net charge of the protein and to reach the same salt concentration as in the cryo-EM study. To correct unfavorable geometry and steric clashes, energy minimization of the system was conducted using the steepest descent algorithm with a force constant of 1000 kJ/mol/nm and a maximum step size of 0.1 Å, and the maximum number of iterations was set to 50000 steps. After the energy minimization, the system was subjected to a 500-ps long canonical ensemble (NVT) equilibration at a target temperature of 300 K using a velocity-rescaling thermostat (Bussi et al. 2007) with a temperature coupling time constant of 0.1 ps. During the NVT equilibration, all heavy atoms were position-restrained with a force constant of 1000 kJ/mol/nm to equilibrate solvent (water and ions) around the protein. After the NVT equilibration, a 5-ns long isothermal-isobaric ensemble (NPT) equilibration was performed with a coupling of the isotropic Parrinello-Rahman barostat (Parrinello and Rahman 1981) at a target pressure of 1 atm (coupling time constant: 2 ps) and a target temperature of 300 K (coupling time constant: 0.1 ps). During the 5-ns NPT equilibration, only the heavy atoms of the protein backbone were position-restrained with a force constant of 1000 kJ/mol/nm to relax protein side-chains and to allow their interactions with solvent. After the NPT equilibration, another NPT equilibration of 5 ns was performed without any position restraints to relax all the atoms. The equilibrated system was submitted to a production run of 200 ns (NPT ensemble) without any position restraints. All steps of the energy minimization, the equilibrations, and the production run were repeated ten times with an assignment of random initial velocities for all the atoms, which were drawn from the Maxwell-Boltzmann distribution at 300 K. All simulations were performed using the Amber99SB*-ILDN force field (Best and Hummer 2009; Lindorff-Larsen et al. 2012). The neighboring particle search was carried out with a time interval of 20 fs for the calculation of non-bonded pair forces with a cut-off radius of 1 nm. Periodic boundary conditions (PBCs) were applied over the simulation time to avoid artifacts from the finite size of the simulation box. Long-range electrostatic interactions were treated by the particle mesh Ewald (PME) method (Essmann et al. 1995) with a Fourier grid spacing of 1.2 Å. The Lennard-Jones interactions and short-range Coulomb interactions were evaluated with a cut-off radius of 1 nm. All covalent hydrogen bonds of the protein were constrained using the parallel linear constraint solver (P-LINCS) algorithm (Hess 2008). An integration time step of 4 fs, instead of 2 fs, was allowed by an application of the virtual site approach (Feenstra et al. 1999), which removes degrees of freedom from hydrogen atoms in the protein. Coordinates were saved every 10 ps. All simulations were performed using the Gromacs 4.6.7 software package (Pronk et al. 2013). For the calculation of root mean square deviations (RMSDs) of backbone atoms, two exterior subunits were excluded. Hydrogen bond analysis between water and backbone H-N as well as backbone H-N and other residues, which was used to estimate the probability of H/D exchange, were performed using the Gromacs tool *g_hbond*. To avoid any artifacts arising from the limited number of subunits (hexamer), the estimated probability of H/D exchange in the two inner subunits surrounded by neighboring subunits were calculated and selected to represent the polymer structure (Fig. S3).

### Visualization

Data visualizations were carried out using matplotlib (Hunter 2007) with Numpy and Scipy (van der Walt et al. 2011). The cryo-EM structure of the type 1 pilus rod was visualized using PyMOL 1.3 (Schrödinger 2015).

## Results

**Fig. 2:**
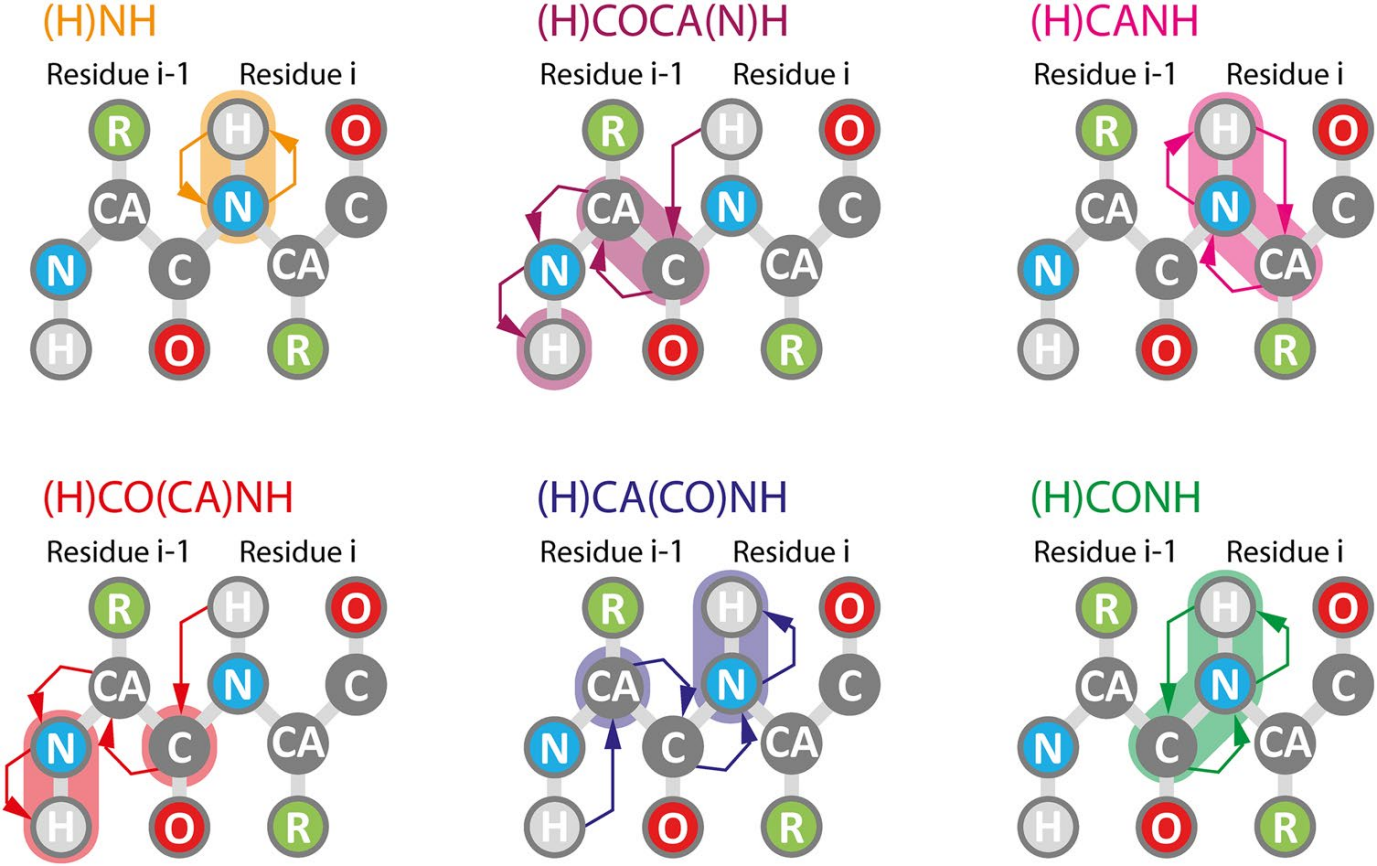
Schematic diagrams of the magnetization transfers. The arrows indicate the direction of the magnetization transfer in (H)NH (orange), (H)COCA(N)H (purple), (H)CANH (magenta), (H)CO(CA)NH (red), (H)CA(CO)NH (blue), and (H)CONH (green) experiments. The shaded regions around the nuclei represent acquisition dimensions of the experiment

In order to obtain chemical shifts of FimA in assembled pili, the following six spectra of the reprotonated sample were acquired using a spectrometer with a ^1^H Larmor frequency of 900 MHz: 2D (H)NH, 3D (H)CANH, 3D (H)CO(CA)NH, and 3D (H)COCA(N)H for intra-residual connectivity and 3D (H)CA(CO)NH and 3D (H)CONH for inter-residual connectivity (Fig. 2). The ^13^C and ^15^N chemical shifts were calibrated to the chemical shifts from the previous ^13^C-detected solid-state NMR experiments (Habenstein et al. 2015) to compensate for the isotopic shift effect induced by the deuterium incorporation (Smith et al. 2017).

**Fig. 3:**
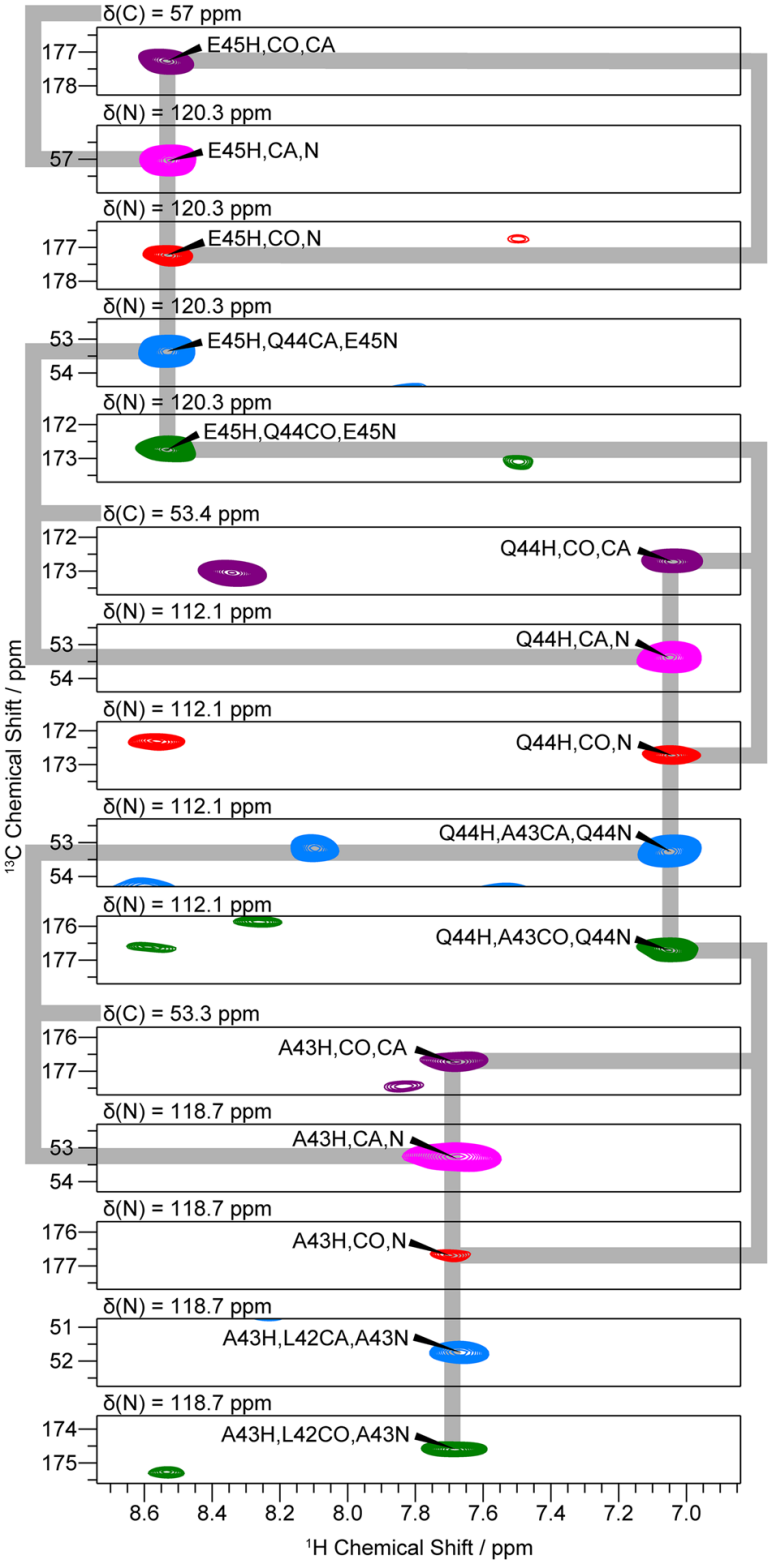
Strip plots of a sequential walk along the backbone of a three-residue stretch from Glu45 to Ala43. The peaks in the strips are color coded as in Fig 2: (H)COCA(N)H in purple, (H)CANH in magenta, (H)CO(CA)NH in red, (H)CA(CO)NH in blue, and (H)CONH in green. The grey lines indicate identical ^1^H or ^13^C chemical shifts. The chemical shift of the z-axis on each strip is annotated above the strip. Inter- and intra-residual correlation peaks of residues Glu45-Ala43 are labeled

We achieved backbone chemical shift assignments (^1^H^N^, ^15^N, ^13^Cα, and ^13^CO) for all residues in FimA except the first three N-terminal residues (Fig. 3 and Table S9), which were not detected due to low efficiency of CP transfer in the highly dynamic N-terminus. In addition, many peak splittings and peak broadenings were detected (Fig. S4 and Table S9), which were not identified in the previous ^13^C-detected solid-state NMR study (Habenstein et al. 2015). The peak splittings and the peak broadenings are mainly found in the region between subunit *i* and subunit *i*-1. We assume that the peak splittings and the peak broadenings are related to conformational heterogeneity (polymorphism) and/or slow conformational dynamics.

The CSPs of backbone amide ^1^H^N^ and ^15^N resonances, calculated from chemical shifts of self-complemented monomeric FimA (FimAa, solution NMR) (Puorger et al. 2011) and polymerized FimA (pili, solid-state NMR), indicate which residues are involved in intermolecular interactions resulting from polymerization (Fig. S2). Residues adjacent to Ala51 of subunit *i*+3 (Gln89-Ser95 of subunit *i*; Val37 and Val52 of subunit *i*+3) and residues adjacent to Ala77 of subunit *i* (Ile78, Ala87, and Leu113 of subunit *i*; Arg106 and Thr107 of subunit *i*+3) show the largest CSPs. While the layer-to-layer subunit interface around Ala51 and Ala77 in pili exhibits a different chemical environment compared to self-complemented monomeric FimA (FimAa), the regions related to DSC are relatively similar (Thr4-Asn18, Val28-Gly35, and Gly146-Tyr158).

**Fig. 4:**
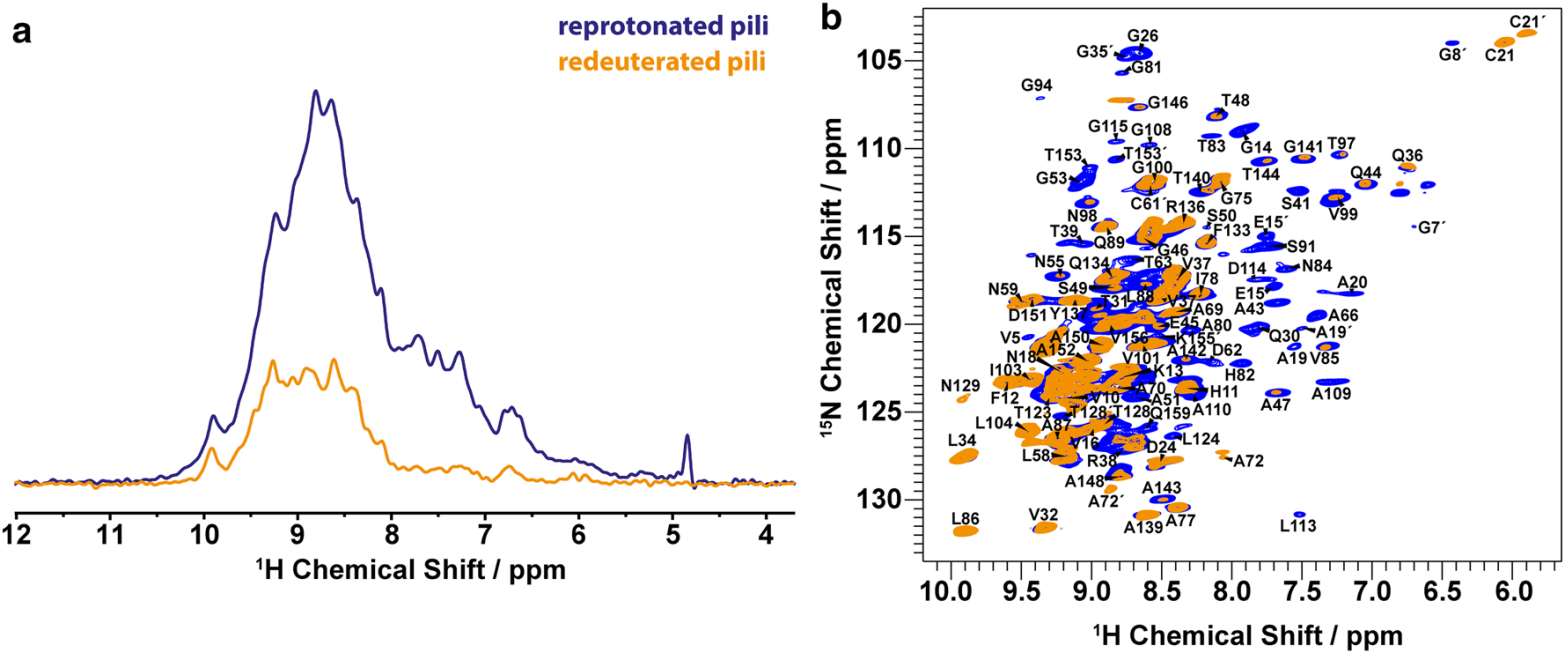
Comparison of proton-detected ^15^N-^1^H correlation spectra of the reprotonated and redeuterated type 1 pili samples recorded using 1.9 mm rotors at 40 kHz MAS and a ^1^H Larmor frequency of 700 MHz. **a** 1D version and **b** 2D version of (H)NH spectral overlays for the reprotonated (blue) and redeuterated (orange) pili samples. Peak splitting in the (H)NH 2D (**b**) is denoted by prime symbols. Peaks that are only present in the spectra of reprotonated pili (blue) and not in the spectra of redeuterated pili (orange) indicate residues undergoing fast H/D exchange

H/D exchanged sites of pili were characterized by comparisons of 1D/2D (H)NH and 3D (H)CANH spectra of reprotonated pili and redeuterated pili. We did not observe any chemical shift changes between reprotonated and redeuterated pili (Fig. 4b), which indicates that redeuteration does not affect the structural integrity of the pre-assembled type 1 pili (see also Fig. S5). The vanished peaks in the 2D H-N correlation spectrum of redeuterated pili (orange in Fig. 4b) correspond to fast H/D exchange of backbone amides. A comparison of the intensity in 1D (H)NH spectra between reprotonated pili (blue in Fig. 4a) and redeuterated pili (orange in Fig. 4a) shows that approximately two-third of the amide protons in type 1 pili have undergone catalytic exchange to deuterons.

**Fig. 5:**
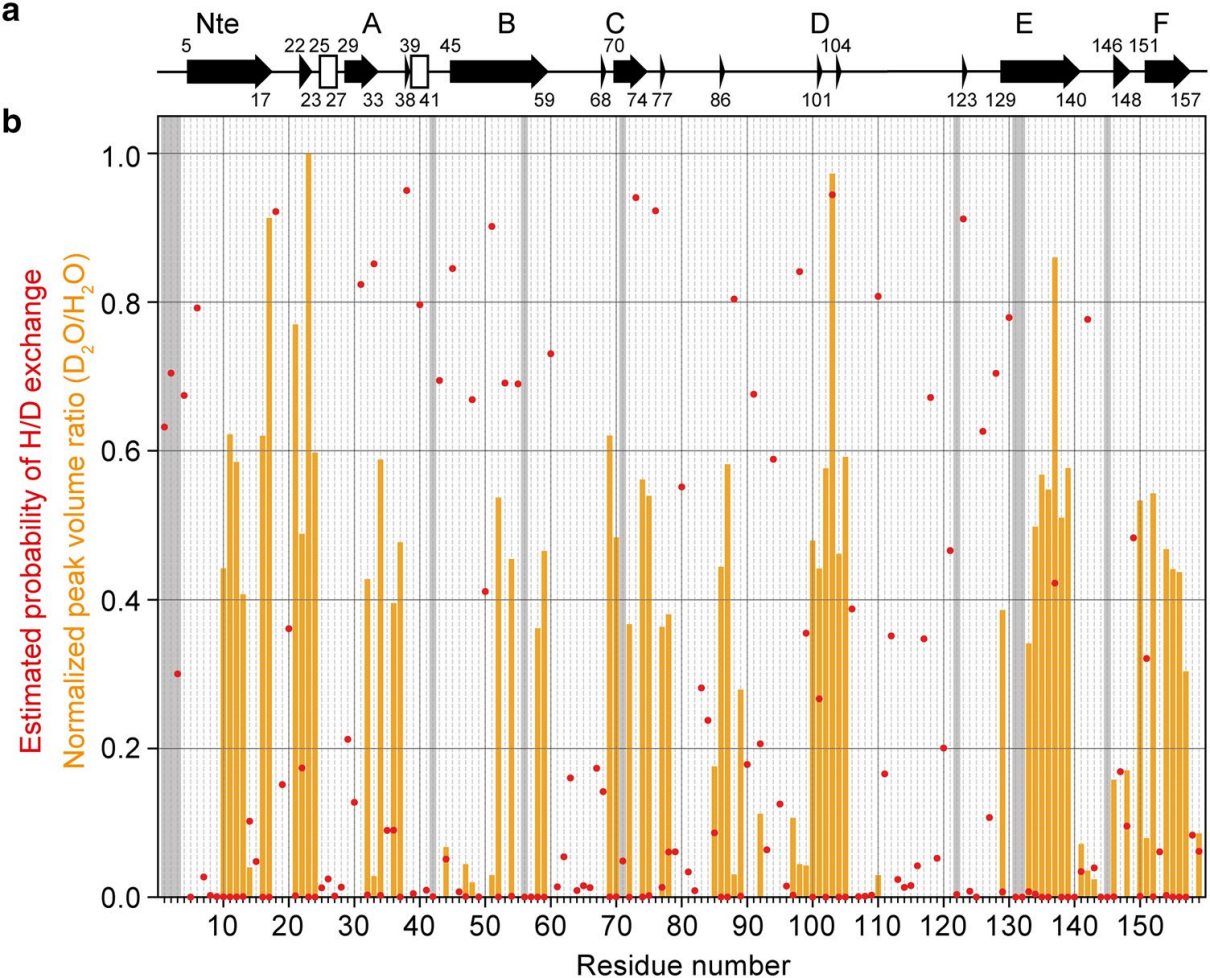
Comparison of experimental H/D exchange and in silico estimations of H/D exchange of the backbone amides of type 1 pili. **a** Secondary structure from the cryo-EM structure (PDB entry 5OH0) (Hospenthal et al. 2017), calculated by the DSSP program (Kabsch and Sander 1983). **b** Normalized peak volume ratios based on 3D (H)CANH spectra of redeuterated and reprotonated pili describing the extent of relative protection of backbone amide protons against H/D exchange. Missing data points are represented by grey shade: the two proline residues (Pro132 and Pro145), the undetected N-terminal region (Ala1, Ala2, and Thr3) and overlapping peaks (Leu42, Ile56, Val71, Thr122, and Ile131). Estimated probabilities of H/D exchange based on MD simulations are represented by red dots

**Fig. 6:**
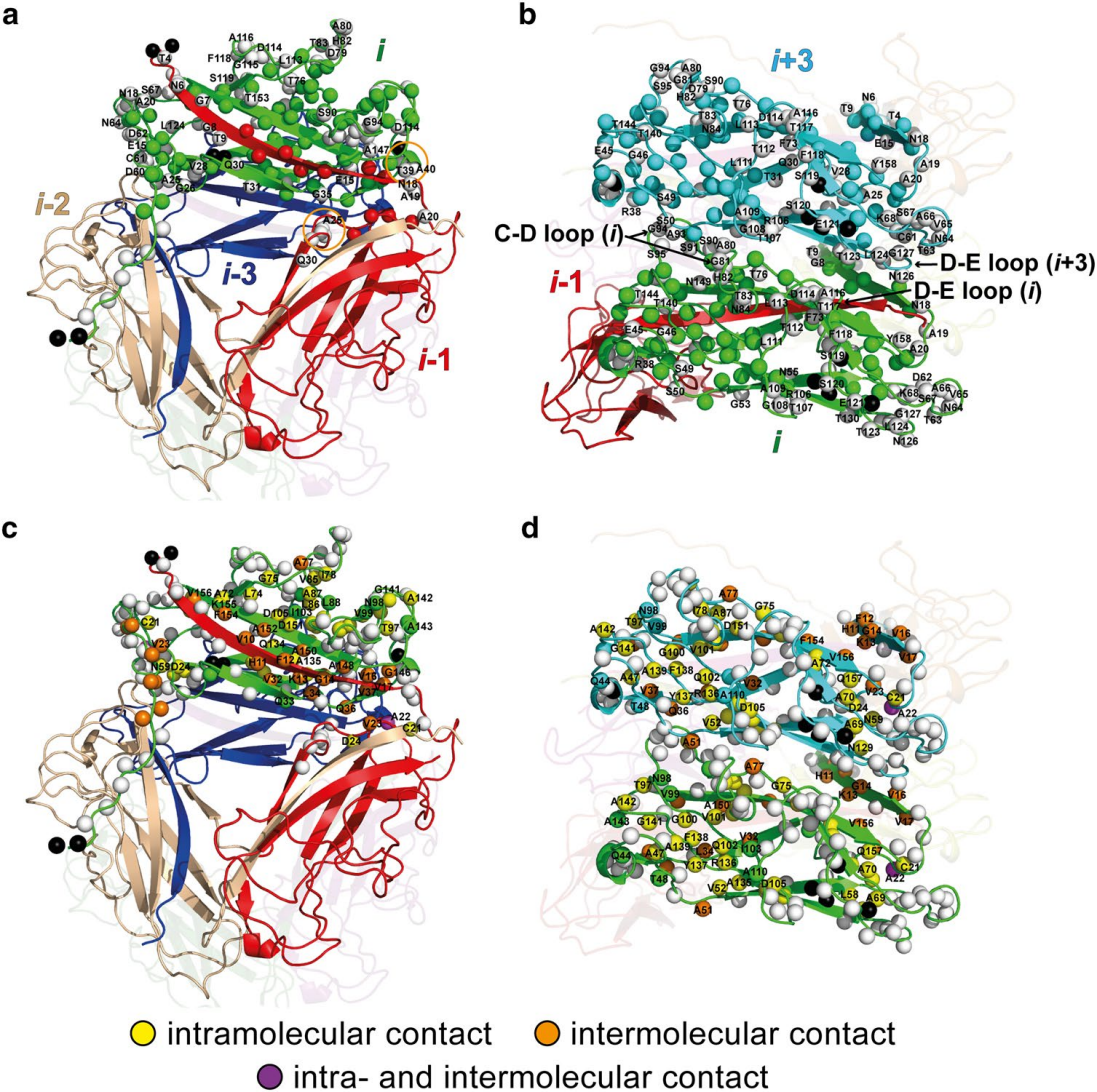
H/D exchange of backbone amide protons in the type 1 pilus rod plotted onto the cryo-EM structure (PDB entry 5OH0) (Hospenthal et al. 2017). Redeuterated sites are represented by white spheres. **a** For clarity only subunit *i* (green) and the N-terminal part of subunit *i*−1 (red) are shown as sphere models. The two α-helices (Ala25–Ser27 and Thr39–Ser41), which are completely exposed to H/D exchange, are indicated by orange circles. **b** The highly solvent accessible interface of subunit *i* (green) and subunit *i*+3 (light blue) is annotated (C-D loop and D-E loop). **c** and **d** The protected amide protons in subunit *i* and subunit *i*−1 (**c**) and in subunit *i* and subunit *i*+3 (**d**) are categorized into intramolecular (yellow spheres) or intermolecular (orange spheres) contacts (Fig. S6). Residues that are involved in both intra- and intermolecular contacts are shown as purple spheres and residues for which data is missing are shown as black spheres

To accurately quantify residue-specific deuterium incorporation, we extracted peak intensities from 3D (H)CANH spectra of reprotonated and redeuterated pili, which was not possible in 2D H-N correlation spectra due to peak overlap. The H/D exchange pattern, represented as the normalized ratio of peak intensities between redeuterated and reprotonated type 1 pili in Fig. 5b (orange bars), shows that the surface-exposed regions (Thr4, Asn6, Asn18–Ala20, Val28–Thr31, Ala43, Glu45–Gly46, Ser49, Asn55, Asp60–Lys68, Gly81–Asn84, and Leu111–Ser119) have, as expected, completely undergone H/D exchange (Fig. 6). Regions that are protected from H/D exchange should either be buried within a subunit or protected by intersubunit interactions. Regions that are protected from H/D exchange due to intramolecular contacts are shown as yellow spheres in Fig. 6c and d (Cys21-Ala22, Asp24, Ala47, Val52, Phe54, Leu58–Asn59, Ala69–Ala70, Ala72, Leu74–Gly75, Ile78, Val85–Ala87, Gln89, Ala92, Thr97, Gly100–Gln102, Leu104–Asp105, Asn129, Phe133–Ala139, Gly141–Ala142, Asp151, Lys155, and Gln157). Regions that are protected due to intermolecular contacts are shown as orange spheres in Fig. 6c and d and are located between subunits *i* and *i*+3 (Ala51 and Ala77) or between subunits *i* and *i*-1 (Val10–Gly14, Val16–Val17, Ala22–Val23, Val32, Leu34, Gln36-Val37, Gly146, Ala148, Ala150, Ala152, Phe154, and Val156) (see Fig. S6 for a summary of intra- and intermolecular contacts).

There are, however, some amide protons that are completely exposed to H/D exchange despite being involved in intramolecular (Gly35, Thr76, Asp79, Ser90, Ala96, Thr107-Ala109, Asn125, Gly127, Thr140, Thr144, Asn149, and Thr153) or intermolecular (Val5, Gly7–Thr9, Glu15, Ser90, Ser95, and Tyr158) contacts. Interestingly, the two α-helices located in the interface of subunits *i* and *i*-1 (Ala25–Ser27 and Thr39–Ser41) are completely exposed to H/D exchange (Fig. 6). This may be related to polymorphism and/or slow conformational dynamics in the interface between subunits *i* and *i*−1 (Fig. S4b and Table S9).

In the donor strand, the amide protons of residues Val10–Lys13 and Val16-Val17 including Val10, Phe12, and Val16 (P2, P3, and P5, respectively) are highly protected while Gly8 (P1) is exposed to solvent (Fig. 6a). This indicates that the second half of the donor strand is tightly bound as in a β-sheet, whereas the first nine N-terminal residues are exposed to solvent. In the second half of the donor strand, Gly14 (P4) is relatively strongly exposed to H/D exchange. Mutation of the highly conserved Gly14 of P pili to Asn (Gly14Asn) showed impairment of self-polymerization of P pili (Verger et al. 2007). The mutation may hamper binding of the neighboring hydrophobic P residues (Phe12 and Val16) by an increased steric hindrance of Gly14Asn.

The most protected amide proton is at Val23, which is located in the highly protected short β-strand region of subunit *i*-1 (residues Cys21-Asp24) between the donor strand and the pilin body. This region is adjacent to the other three subunits (subunits *i*, *i*−2, *i*−3 in Fig. 6a) and is complemented with a highly conserved intramolecular disulfide bond between Cys21 and Cys61. Spaulding et al. reported that mutation of Ala22 to Arg resulted in lowering the unwinding force in force response versus elongation from an optical tweezer experiment (Spaulding et al. 2018), no intracellular bacterial communities (IBCs), and reduction of the ability to invade mouse bladder cells. In the FimA-FimC complex structure, the short β-strand (residues Ala22–Ala25) of FimA interacts with FimC (Crespo et al. 2012). Therefore, the H/D exchange protected short β-strand region may contribute to the mechanical properties and function of the type 1 pilus rod.

In contrast, part of the loop region between β-strands C and D (C-D loop: residues Gly75–Gly100) and the loop region between β-strands D and E (D-E loop: residues Asp105–Thr128) exhibit a high level of H/D exchange (Fig. 5a and b). In the pilus rod, these loops are located in the layer-to-layer subunit interface and on the exterior surface (Fig. 6b). The number of layer-to-layer subunit interactions per helical turn is more than three and these interactions are repeating along the axis of the pilus rod. The H/D exchange and structure of the layer-to-layer subunit interface may explain the elongation-competent property of these regions under external shear stress, which distribute the tension along the pilus rod.

We also performed MD simulations to study the dynamics of the pilus rod. Ten 200 ns MD simulations were carried out, resulting in total in a 2 µs trajectory. To check deviations from the starting structure and integrity of MD systems, the root mean square deviations (RMSDs) of the backbone of the inner four pilus rod subunits were calculated (Fig. S7). RMSDs of all ten MD runs converged to less than 2 Å, indicating that systems were well equilibrated over the simulation time. To estimate the probability of H/D exchange for backbone amide protons from the MD simulations, the number of hydrogen bonds between water and backbone H-N in the absence of hydrogen bonds between the backbone H-N and other residues was calculated (red dots in Fig. 5b). Regions with no or relatively few hydrogen bonds between backbone amide protons and water mostly correspond to the slow H/D exchange sites (orange bars in Fig. 5b) with the exception of Ile103 and Tyr137, where large discrepancies were observed. This may be due to the fact that experiments and simulations were performed at different time scales, i.e. the MD simulation time of 2 µs versus the H/D exchange of several hours.

## Discussion

In the present study, we assigned the backbone chemical shifts (residues Thr4-Gln159) of perdeuterated, fully proton back-exchanged pili based on multi-dimensional NMR spectra acquired by proton-detected solid-state NMR under fast MAS at 40 kHz (Table S9). To investigate H/D exchange of type 1 pili, we employed deuterium back-exchange of a perdeuterated, fully proton back-exchanged sample (Gallagher et al. 1992; Whittemore et al. 2005; Wang et al. 2011; Medeiros-Silva et al. 2017; Grohe et al. 2017; Medeiros-Silva et al. 2016; Chevelkov et al. 2017). This approach is different in the earlier steps before the redeuteration from the inversely fractional deuteration (iFD) (Medeiros-Silva et al. 2016). Our pili sample was uniformly [^2^H,^13^C,^15^N]-labeled during expression, proton back-exchanged at all labile sites by purification in 100% H_2_O buffer under denaturing conditions, followed by pili assembly in non-denaturing 100% H_2_O buffer. We applied this method to a supramolecular assembly while the iFD method has been used previously for membrane proteins. This H/D exchange study of pre-assembled type 1 pili revealed the distinct pattern of H/D exchange in the head-to-tail and the layer-to-layer regions. The regions that are highly protected from H/D exchange are located in the latter part of the donor strand (residues Val10–Lys13 and residues Val16–Val17) and the short β-strand (residues Cys21–Asp24) between the donor strand and the pilin body. From the mutational study by Spaulding et al., we can infer that the substitution of Ala22 to the basic and bulky residue Arg may influence the highly conserved intramolecular disulfide bond of Cys21–Cys61 and tight helical packing of subunits by steric clashes. In the head-to-tail interface, the two α-helices (Ala25–Ser27 and Thr39–Ser41) are completely exposed to H/D exchange (Fig. 5b). The exposure of the amide protons in the α-helices may be related to polymorphism and/or slow conformational changes as found from peak splittings in the NMR spectra for the head-to-tail interface (Fig. S4). The layer-to-layer subunit interactions are more prone to break based on the fact that most of the interface is composed of loops and is highly exposed to H/D exchange. Here we found that only two backbone amide protons (Ala51 and Ala77) are protected by layer-to-layer intermolecular contacts. The CSPs between the self-complemented monomeric FimA (solution NMR) (Puorger et al. 2011) and pili (solid-state NMR) show that there are significant differences of chemical environments in the vicinity of Ala51 and Ala77 (Fig. S2) that indicate a small layer-to-layer interface in pili and/or their conformational dynamics. In contrast, many amide protons are protected from H/D exchange by head-to-tail intermolecular contacts (Val10–Gly14, Val16–Val17, Val23, Val32, Leu34, Gln36–Val37, Gly146, Ala148, Ala150, Ala152, Phe154, and Val156) (Fig. 6c, d). The backbone-mediated intermolecular interactions in the head-to-tail interface may contribute to the extreme stability of pili in addition to their hydrophobic side-chain interactions.

The H/D exchange results from NMR experiments and the estimated probability of H/D exchange based on hydrogen bond dynamics from MD simulations are generally in good agreement. Nevertheless, it is important to note that MD simulations only provide dynamics of the stiff coiled-state of the pilus rod on a relatively short time-scale. In order to further improve consistency with experimental observables, MD simulations using enhanced sampling techniques may be considered in future studies (Bernardi et al. 2015).

## Conclusions

Our integrative approach combining solid-state NMR spectroscopy and MD simulations give atomic resolution insights into mechanical properties and stability of type 1 pili. Investigations of the degree of H/D exchange at deuterium back-exchangeable sites from the perdeuterated, fully proton back-exchanged pili sample reveal the H/D exchange behavior in the context of assembled pili. We found high stability of backbone amides in the head-to-tail interface and the core of monomeric FimA in pili, whereas the layer-to-layer interface and exterior surface areas exhibit low protection against H/D exchange. The different H/D exchange behavior is consistent with resistibility and extensibility of pili under shear force. MD simulations of the pilus rod provided an estimated probability of H/D exchange of backbone H-N by hydrogen bond dynamics. The present study may also give insights into potential binding sites for new types of antibiotics such as *coilicide*, which makes UPEC prone to detach from host cells under bulk flow of urine (Klinth et al. 2012). A multi-drug strategy combining this approach with adhesin-host receptor inhibitors (Hartmann et al. 2011; Totsika et al. 2013) and *pilicide*, which blocks the interaction between chaperone-subunit and usher (Pinkner et al. 2006), may be a conceivable alternative to treat patients with urinary tract infection in the future.

## Electronic supplementary material

Below is the link to the electronic supplementary material.

Supplementary file1 (PDF 1344 kb)
