# Supplementary material for: Characterization of H/D exchange in type 1 pili by proton-detected solid-state NMR and molecular dynamics simulations
Source: J Biomol NMR. 2019 Apr 26;73(6):281–91. doi: 10.1007/s10858-019-00247-3 (PMC6692446; doi:10.1007/s10858-019-00247-3)
Supplement: Supplementary file 1 — Supplementary file1 (PDF 1344 kb) [file 10858_2019_247_MOESM1_ESM.pdf]

Supporting Information for

Characterization of H/D Exchange in Type 1 Pili by Proton-Detected Solid-State NMR and Molecular Dynamics Simulations

Songhwan Hwang[1], Carl Öster[1], Veniamin Chevelkov[1], Karin Giller[2], Sascha Lange[1], Stefan Becker[2], and Adam Lange*[1,3]

[1]Department of Molecular Biophysics, Leibniz-Forschungsinstitut für Molekulare Pharmakologie (FMP), Berlin, Germany

[2]Department of NMR-based Structural Biology, Max Planck Institute for Biophysical Chemistry, Göttingen, Germany

[3]Institut für Biologie, Humboldt-Universität zu Berlin, Berlin, Germany

*To whom correspondence should be addressed. Telephone: +49 30 947 93 190. E-mail: alange@fmp-berlin.de.

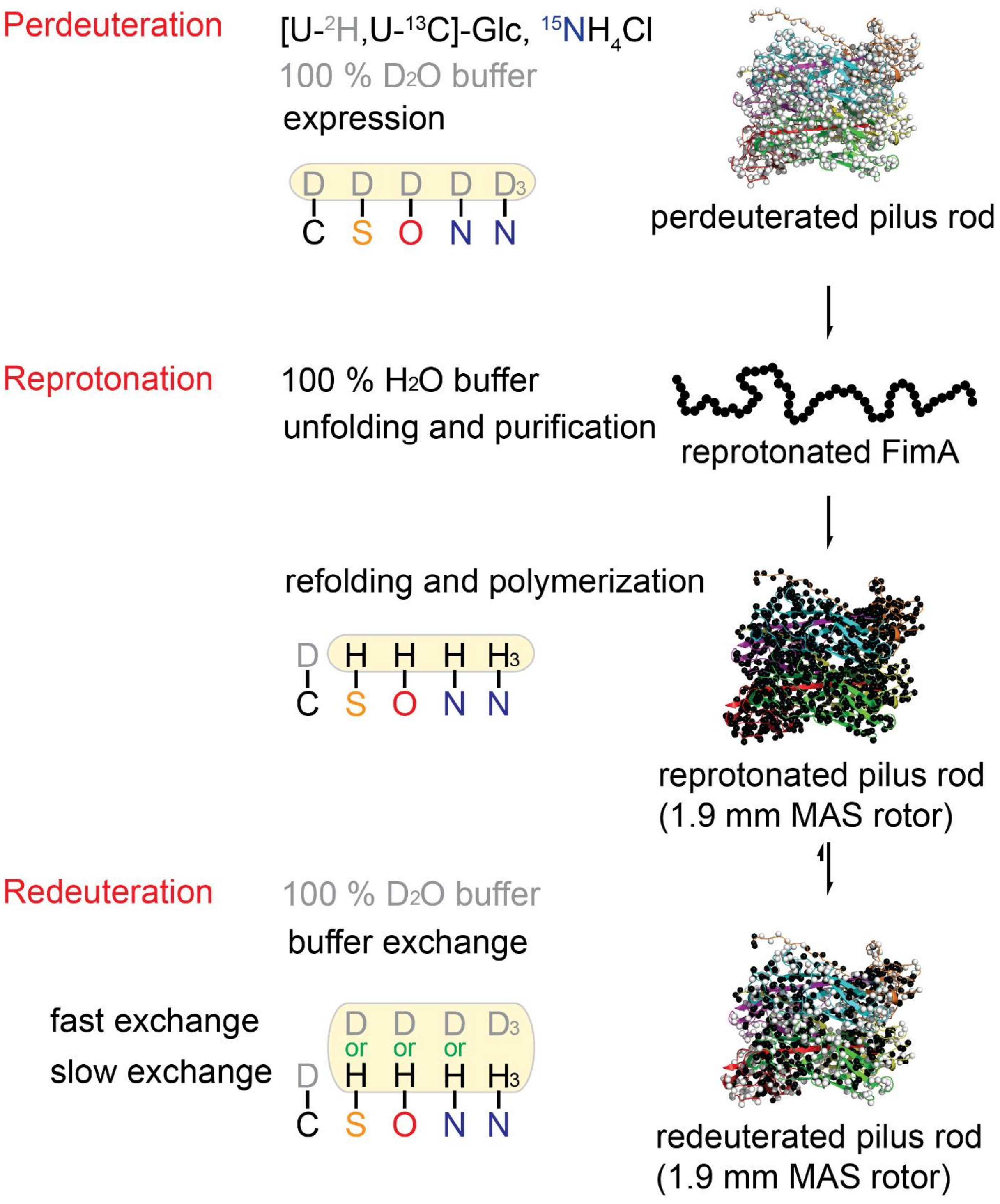

Fig. S1 Schematic diagram of the sample preparation process.

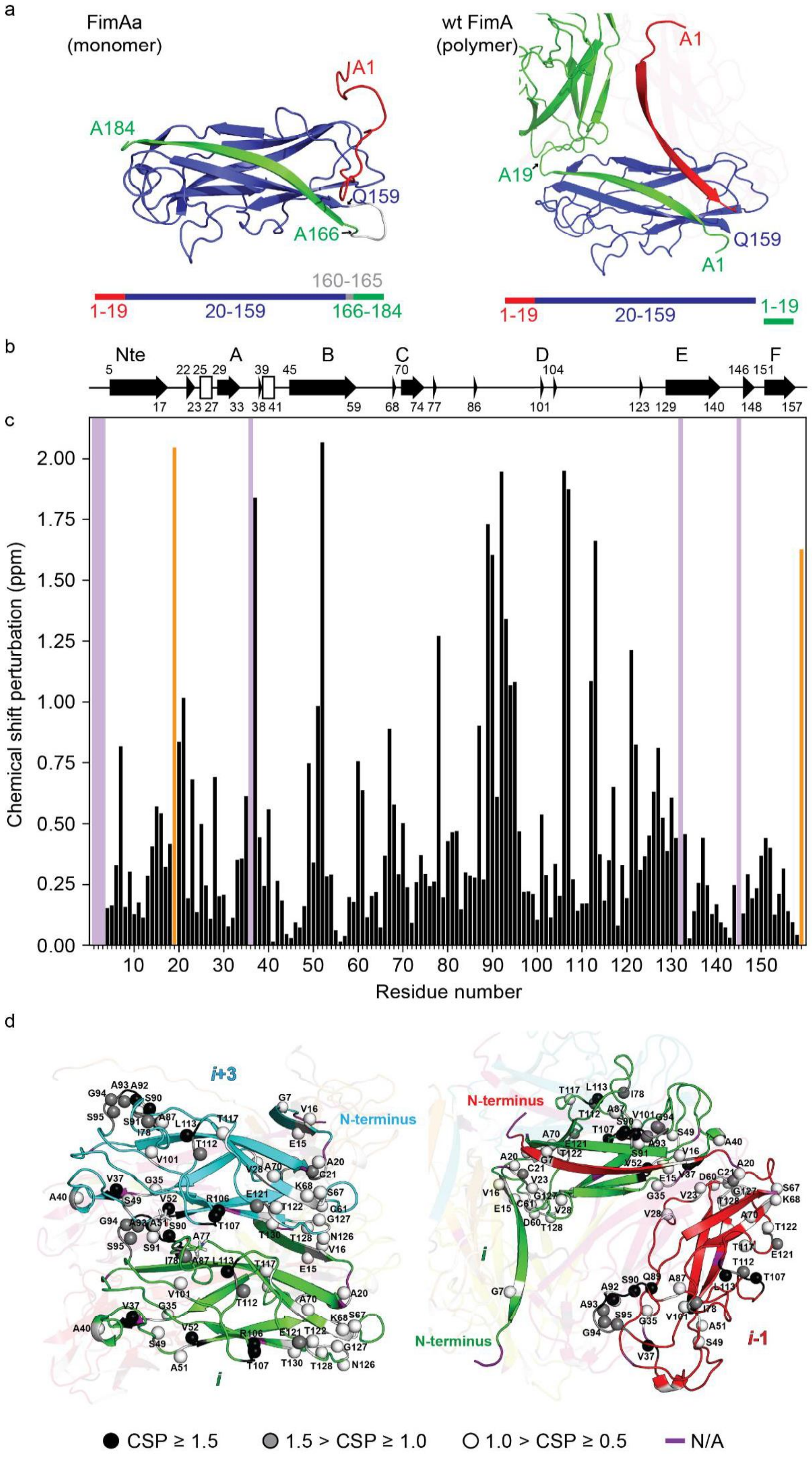
a
FimAa
(monomer)
wt FimA
(polymer)
A1
A184
Q159
A166
160-165
1-19
20-159
166-184
A19
A1
Q159
A1
1-19
20-159
1-19
b
Nte
A
B
C
D
E
F
c
Chemical shift perturbation (ppm)
Residue number
d
i+3
N-terminus
i
N-terminus
i-1
N-terminus
CSP ≥ 1.5
1.5 > CSP ≥ 1.0
1.0 > CSP ≥ 0.5
N/A

Fig. S2 Chemical shift perturbations (CSPs) of backbone amide $^{1}H^{N}$ and ^{15}N of FimA upon polymerization. (a, left panel) The structure of self-complemented monomeric FimAa by solution NMR (PDB entry 2JTY)[1], where the self-complemented donor strand (Ala166-Ala184 correspond to Ala1-Ala19 in wild-type FimA) is attached to the C-terminus of wild-type FimA with an intervening six-glycine linker (Gly160-165). (a, right panel) The structure of the type 1 pilus rod determined by cryo-EM (PDB entry 5OH0)[2]. Note that the complemented donor strand (Ala1-Ala19, colored green) is from the neighboring subunit. (b) Secondary structure based on the cryo-EM structure of type 1 pili, calculated by the DSSP program[3]. (c) CSPs for type 1 pili calculated from the backbone amide $^{1}H^{N}/^{15}N$ chemical shifts obtained by solid-state NMR (the present study) and $^{1}H^{N}/^{15}N$ chemical shifts of self-complemented monomeric FimAa obtained by solution NMR (PDB entry 2JTY)[1]. For the calculation of the CSPs, chemical shifts of residues Ala1-Ala19 of FimAa (solution NMR) have been substituted with the chemical shifts of residues Ala166-Ala184 of FimAa representing the complemented donor strand. The CSPs of Ala19 and Gln159 (orange bars) may be artefacts due to the different environments used in the solution and solid state studies. Residues for which no CSPs are available, the two prolines (Pro132 and Pro145) and the un-assigned residues (Ala1, Ala2, Thr3, and Gln36), are represented by purple bars. (d) The CSPs are mapped on subunit *i* and subunit *i*+3 (d, left panel) and subunit *i*-1 and subunit *i* (d, right panel) of the type 1 pilus rod (PDB entry 5OH0)[2]. The side chains of the residues protected against H/D exchange in between subunits *i* and *i*+3 (Ala51 and Ala77) are indicated with stick models (d, left panel). Residues with CSPs between 0.5 - 1 ppm are represented by white spheres, residues with CSPs between 1 - 1.5 ppm by grey spheres and residues with CSPs ≥ 1.5 ppm by black spheres.

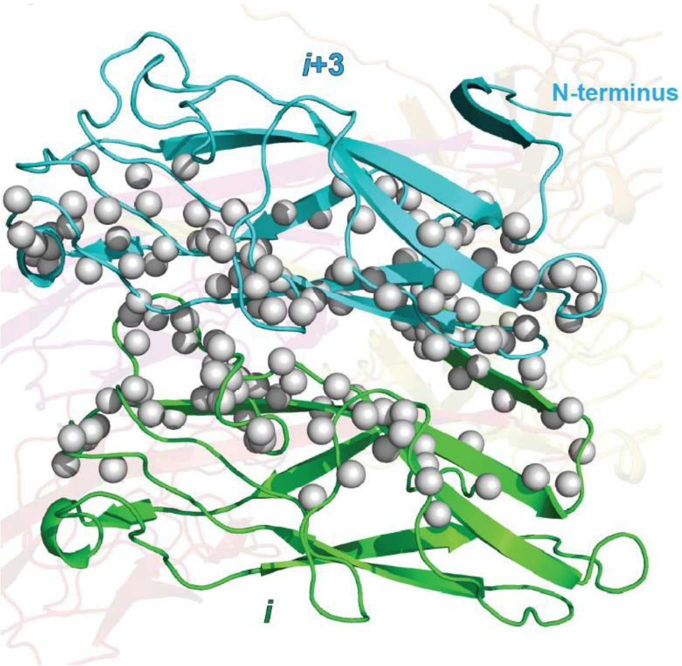

Fig. S3 Distribution of amide protons in the two inner subunits in the six-subunit model used for MD simulations. A six subunit model is used to accurately represent each amide proton in a fully assembled pilus rod. This model, based on the cryo-EM structure of the type 1 pilus rod (PDB entry 5OH0)[2], represents a monomer surrounded by neighboring subunits. The residues in the two inner subunits are selected as follows: Ala1-Ala20 (subunit *i*), Cys21-Ala70 (subunit *i*+3), Val71-Val99 (subunit *i*), Gly100-Ala110 (subunit *i*+3), Leu111-Phe118 (subunit *i*), Ser119-Thr140 (subunit *i*+3), and Gly141-Gln159 (subunit *i*). The selected amide protons are depicted as white spheres.

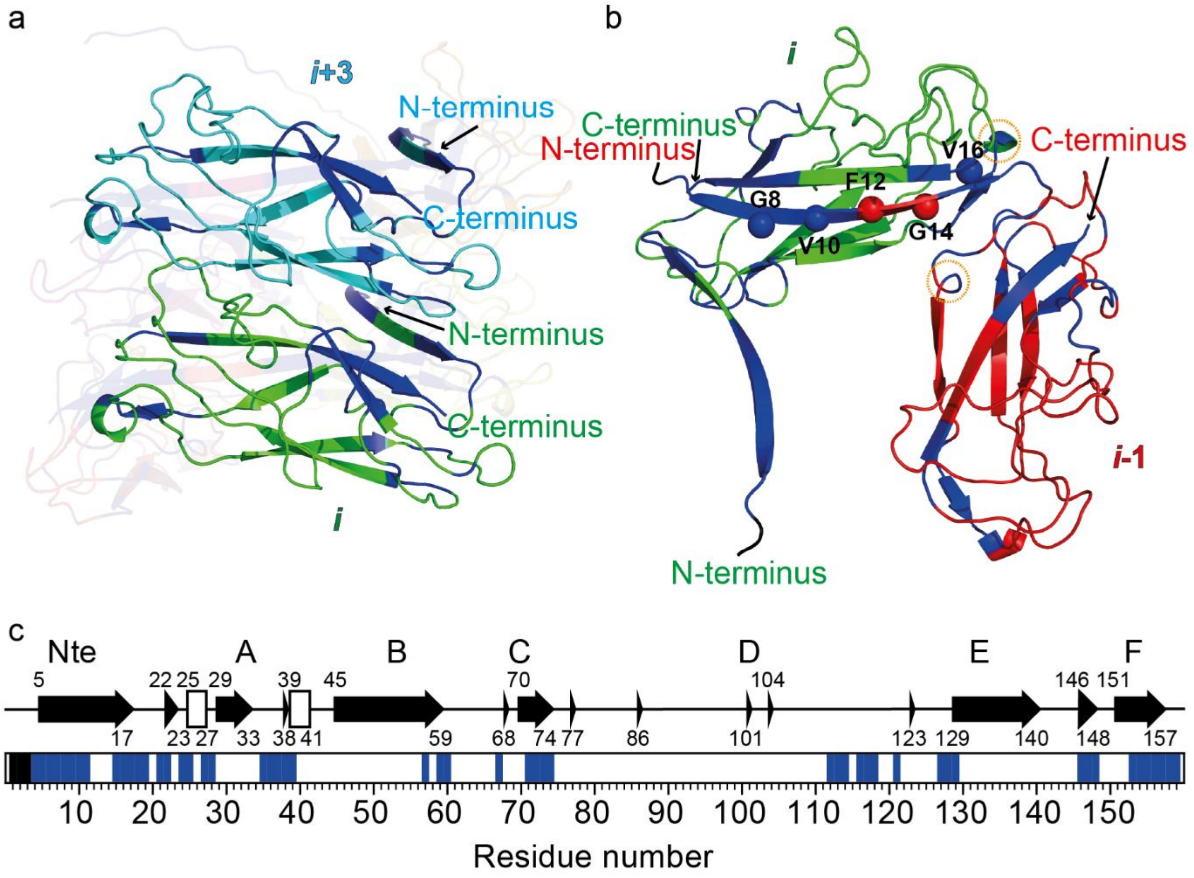

Fig. S4 Peak splitting in type 1 pili. The peak splitting pattern (colored in blue) is mapped on subunit *i* (green) and subunit *i*+3 (light blue) (a) and on subunit *i* (green) and *i*-1 (red) (b) of the type 1 pilus rod (PDB entry 5OH0)[2]. The P1-P5 residues (Gly8, Val10, Phe12, Gly14, and Val16) are shown as filled spheres in (b). The un-assigned residues at the N-terminus are indicated in black. The two α-helices (Ala25-Ser27 and Thr39-Ser41), which are completely exposed to H/D exchange, are highlighted by orange dotted circles. (c, upper panel) Secondary structure of the cryo-EM structure, calculated by the DSSP program[3]. (c, lower panel) Peak splitting pattern plotted as a function of residue number. The peak splittings shown here were detected in the reprotonated pili sample.

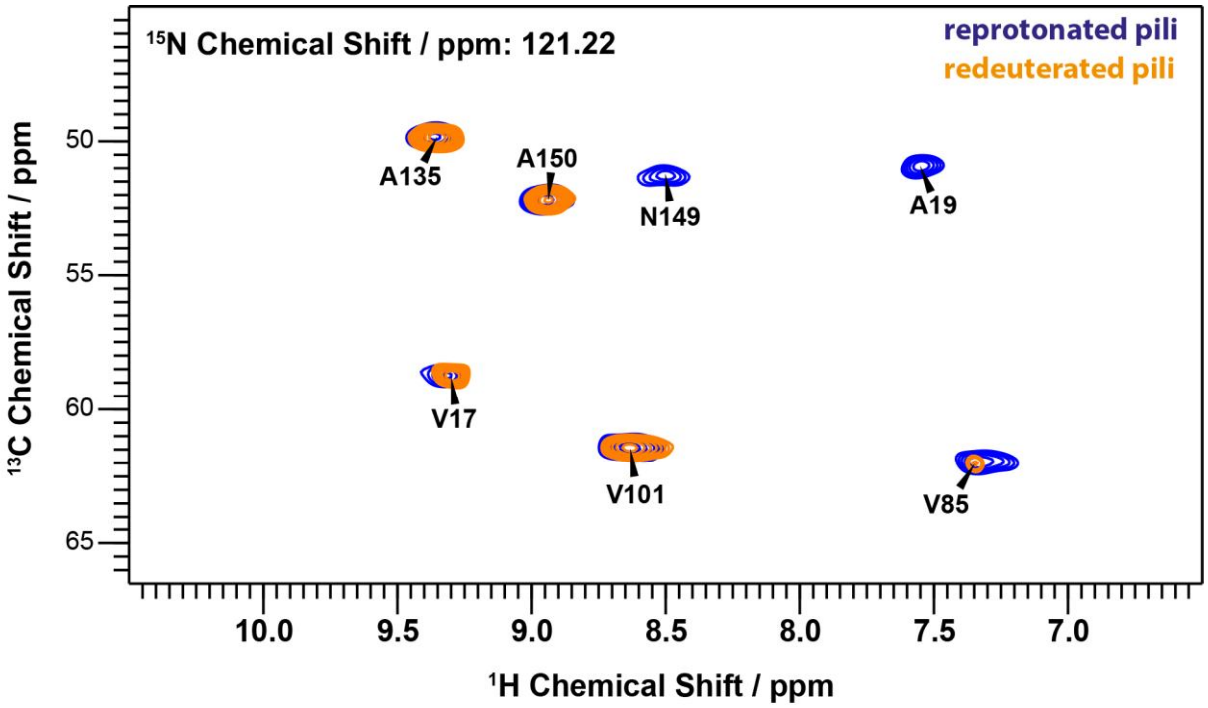

Fig. S5 Comparison of 2D HC planes from 3D (H)CANH spectra of reprotonated pili (blue) and redeuterated pili (orange).

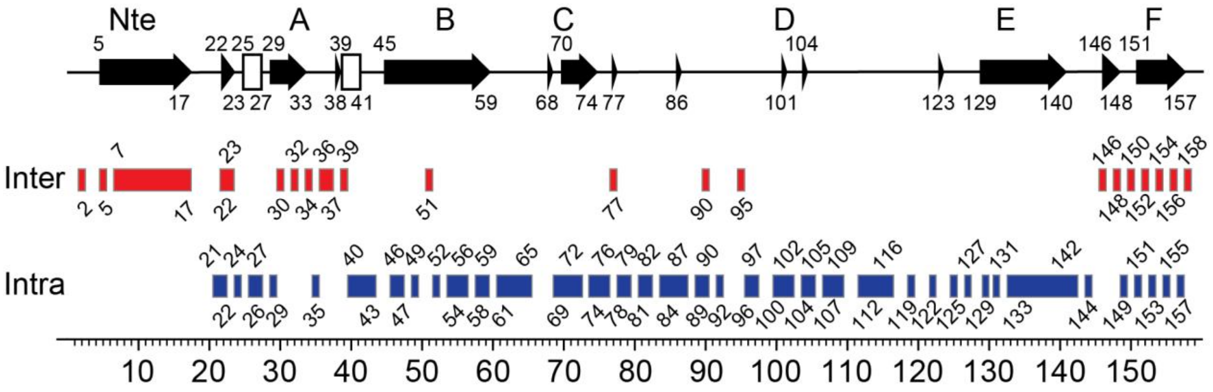

Fig. S6 Schematic diagram of intermolecular (middle panel) and intramolecular (lower panel) contacts in type 1 pili. Secondary structure (upper panel) of the cryo-EM structure (PDB entry: 5OH0)[2], calculated by the DSSP program[3]. The molecular contacts in the cryo-EM structure were calculated with a cut-off radius of 3 Å from backbone amide protons. For the calculation of intramolecular contacts, sequential residues (the preceding and the succeeding residues) are excluded.

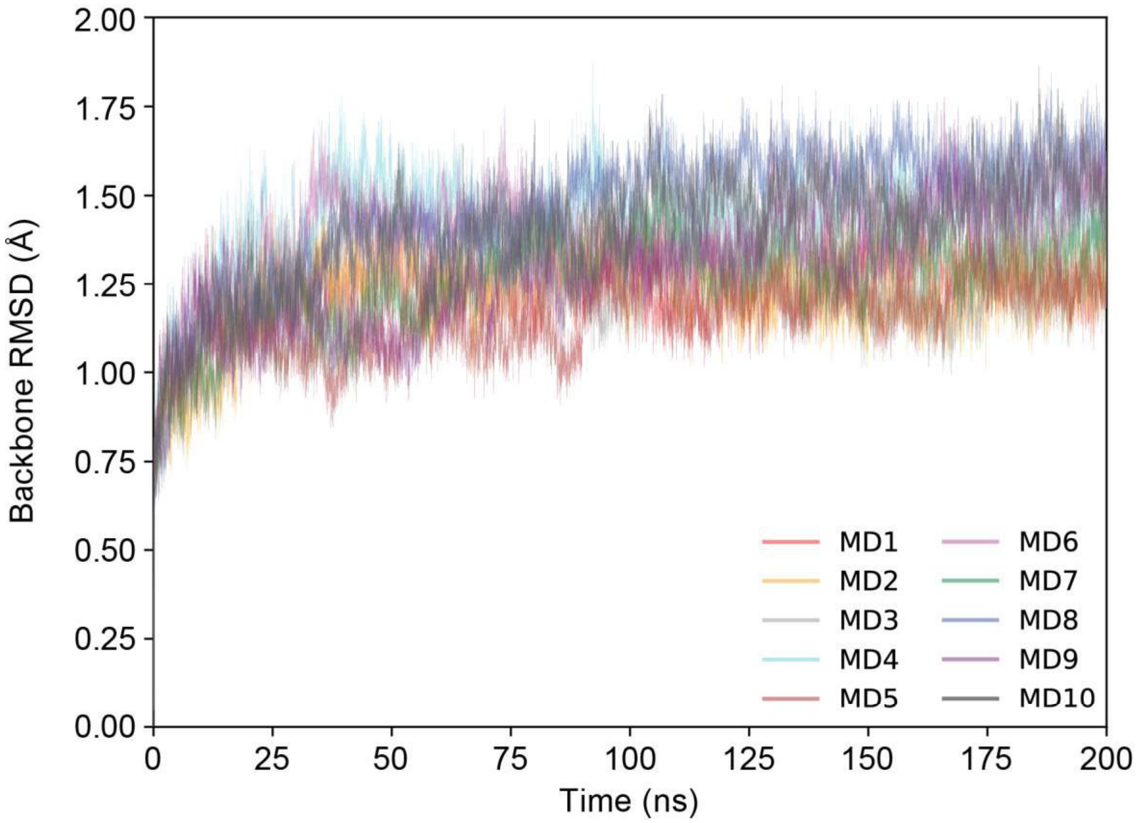

Fig. S7 Root mean square deviations of the backbone atoms of the type 1 pilus rod during 200-ns molecular dynamics simulations.

Table S1 Set of pulse program parameters used for the acquisition of the 2D ^{1}H-^{15}N correlation spectrum of reprotonated pili at 900 MHz ^{1}H Larmor frequency and 40 kHz magic-angle spinning. The radio frequency (r.f.) power values were only determined for the high-power pulses via nutation experiments, the other r.f. powers are calculated assuming a linear amplifier and are therefore only estimates. The employed experiment with corresponding pulse sequence is described in detail in the literature[4,5].

**Parameter**	**Reprotonated pili**
Recycle delay	
Recycle delay	1 s
90° initial ^{1}H excitation pulse	
R.f. power	83.33 kHz
Duration	3 µs
^{1}H-^{15}N CP step	
^{1}H r.f. power	73.55 kHz
^{15}N r.f. power	25.75 kHz
Ramp shape	80-100 % on ^{1}H
Duration	750 µs
^{15}N evolution time	
WALTZ-16 r.f. power on ^{1}H	7.61 kHz
WALTZ-16 pulse duration	50 µs
180° XY r.f. power on ^{13}C	54.32 kHz
180° XY r.f. pulse duration	9.5 µs
180° XY delay	0.5 ms
Maximum acquisition time	24 ms
90° ^{15}N flip pulses	
R.f. power	35.71 kHz
Duration	7 µs
Water suppression	
T delay	77 ms
Spoil pulse duration	1.2 ms
Spoil r.f. power	80.64 kHz
Spoil ramp shape	50-100 %
First pulse duration in train	33 ms
Second pulse duration in train	49.5 ms
Train r.f. power	17.04 kHz
Loops through train (*n*)	2
^{15}N-^{1}H CP step	
^{15}N r.f. power	25.75 kHz
^{1}H r.f. power	73.55 kHz
Ramp shape	100-80 % on ^{1}H
Duration	420 µs
Spin-echo step	
^{1}H pulse r.f. power	83.33 kHz
^{1}H pulse duration	6 µs
Acquisition	
WALTZ-16 r.f. power on ^{15}N	5.08 kHz
WALTZ-16 pulse duration	60 µs
Acquisition time	45 ms
Experimental time	
Experimental time	4 hours

Table S2 Set of pulse program parameters used for the acquisition of the 3D (H)CANH spectrum of reprotonated pili at 900 MHz ^{1}H Larmor frequency and 40 kHz magic-angle spinning. The radio frequency (r.f.) power values were only determined for the high-power pulses via nutation experiments, the other r.f. powers are calculated assuming a linear amplifier and are therefore only estimates. The employed experiment with corresponding pulse sequence is described in detail in the literature[4,5].

**Parameter**	**Reprotonated pili**
Recycle delay	
Recycle delay	1 s
90° initial ^{1}H excitation pulse	
R.f. power	83.33 kHz
Duration	3 µs
^{1}H-^{13}CA CP step	
^{1}H r.f. power	75.83 kHz
^{13}CA r.f. power	30.90 kHz
Ramp shape	80-100 % on ^{1}H
Duration	6 ms
^{13}CA evolution time	
XiX r.f. power on ^{1}H	8.41 kHz
XiX pulse duration	200 µs
^{15}N inversion r.f. power	35.71 kHz
^{15}N inversion pulse duration	14 µs
Maximum acquisition time	5.8 ms
^{13}CA-^{15}N CP step	
^{13}CA r.f. power	26.00 kHz
^{15}N r.f. power	14.82 kHz
Ramp shape	80-100 % on ^{15}N
Duration	9 ms
^{15}N evolution time	
WALTZ-16 r.f. power on ^{1}H	7.94 kHz
WALTZ-16 pulse duration	50 µs
180° XY r.f. power on ^{13}C	54.32 kHz
180° XY r.f. pulse duration	9.4 µs
180° XY delay	0.5 ms
Maximum acquisition time	12.6 ms
90° ^{15}N flip pulses	
R.f. power	35.71 kHz
Duration	7 µs
Water suppression	
T delay	77 ms
Spoil pulse duration	1.9 ms
Spoil r.f. power	84.10 kHz
Spoil ramp shape	100-50 %
First pulse duration in train	40 ms
Second pulse duration in train	68 ms
Train r.f. power	14.96 kHz
Loops through train (n)	2
^{15}N-^{1}H CP step	
^{15}N r.f. power	28.73 kHz
^{1}H r.f. power	74.53 kHz
Ramp shape	100-80 % on ^{1}H
Duration	420 µs
Spin-echo step	
^{1}H pulse r.f. power	83.33 kHz
^{1}H pulse duration	6 µs
Acquisition	
WALTZ-16 r.f. power on ^{15}N	5.70 kHz
WALTZ-16 pulse duration	100 µs
Acquisition time	35 ms
Experimental time	
Experimental time	42.5 hours

Table S3 Set of pulse program parameters used for the acquisition of the 3D (H)CONH spectrum of reprotonated pili at 900 MHz ^{1}H Larmor frequency and 40 kHz magic-angle spinning. The radio frequency (r.f.) power values were only determined for the high-power pulses via nutation experiments, the other r.f. powers are calculated assuming a linear amplifier and are therefore only estimates. The employed experiment with corresponding pulse sequence is described in detail in the literature[4,5].

**Parameter**	**Reprotonated pili**
Recycle delay	
Recycle delay	1 s
90° initial ^{1}H excitation pulse	
R.f. power	83.33 kHz
Duration	3 µs
^{1}H-^{13}CO CP step	
^{1}H r.f. power	72.00 kHz
^{13}CO r.f. power	26.91 kHz
Ramp shape	80-100 % on ^{1}H
Duration	6 ms
^{13}CO evolution time	
XiX r.f. power on ^{1}H	8.41 kHz
XiX pulse duration	200 µs
^{15}N inversion r.f. power	35.71 kHz
^{15}N inversion pulse duration	14 µs
Selective ^{13}C r.f. power	9.38 kHz
Selective ^{13}C pulse duration	400 µs
Selective ^{13}C pulse shape	Gauss cascade Q3
Maximum acquisition time	11.2 ms
^{13}CO-^{15}N CP step	
^{13}CO r.f. power	24.26 kHz
^{15}N r.f. power	17.41 kHz
Ramp shape	80-100 % on ^{15}N
Duration	9 ms
^{15}N evolution time	
WALTZ-16 r.f. power on ^{1}H	9.44 kHz
WALTZ-16 pulse duration	50 µs
180° XY r.f. power on ^{13}C	54.32 kHz
180° XY r.f. pulse duration	9.4 µs
180° XY delay	1 ms
Maximum acquisition time	12.6 ms
90° ^{15}N flip pulses	
R.f. power	35.71 kHz
Duration	7 µs
Water suppression	
T delay	77 ms
Spoil pulse duration	1.2 ms
Spoil r.f. power	84.10 kHz
Spoil ramp shape	100-50 %
First pulse duration in train	55 ms
Second pulse duration in train	93.5 ms
Train r.f. power	15.66 kHz
Loops through train (n)	2
^{15}N-^{1}H CP step	
^{15}N r.f. power	26.97 kHz
^{1}H r.f. power	74.10 kHz
Ramp shape	100-80 % on ^{1}H
Duration	450 µs
Spin-echo step	
^{1}H pulse r.f. power	83.33 kHz
^{1}H pulse duration	6 µs
Acquisition	
WALTZ-16 r.f. power on ^{15}N	5.70 kHz
WALTZ-16 pulse duration	100 µs
Acquisition time	33 ms
Experimental time	
Experimental time	57.5 hours

Table S4 Set of pulse program parameters used for the acquisition of the 3D (H)COCA(N)H spectrum of reprotonated pili at 900 MHz ^{1}H Larmor frequency and 40 kHz magic-angle spinning. The radio frequency (r.f.) power values were only determined for the high-power pulses via nutation experiments, the other r.f. powers are calculated assuming a linear amplifier and are therefore only estimates. The employed experiment with corresponding pulse sequence is described in detail in the literature[4,5].

**Parameter**	**Reprotonated pili**
Recycle delay	
Recycle delay	1 s
90° initial ^{1}H excitation pulse	
R.f. power	83.33 kHz
Duration	3 µs
^{1}H-^{13}CO CP step	
^{1}H r.f. power	73.17 kHz
^{13}CO r.f. power	28.02 kHz
Ramp shape	80-100 % on ^{1}H
Duration	5.1 ms
^{13}CO evolution time	
XiX r.f. power on ^{1}H	8.45 kHz
XiX pulse duration	200 µs
^{15}N inversion r.f. power	35.71 kHz
^{15}N inversion pulse duration	14 µs
Selective ^{13}C r.f. power	9.38 kHz
Selective ^{13}C pulse duration	400 µs
Selective ^{13}C pulse shape	Gauss cascade Q3
Maximum acquisition time	10.6 ms
^{13}CO-^{13}CA DREAM CP step	
Trim pulse r.f. power	54.32 kHz
Trim pulse duration	3.8 µs
DREAM pulse r.f. power	12.30 kHz
DREAM pulse shape	Ramp 100-80 %
DREAM pulse duration	8.7 ms
^{13}CA evolution time	
XiX r.f. power on ^{1}H	8.45 kHz
XiX pulse duration	200 µs
^{15}N inversion r.f. power	35.71 kHz
^{15}N inversion pulse duration	14 µs
Maximum acquisition time	5.8 ms
^{13}CA-^{15}N CP step	
^{13}CA r.f. power	25.70 kHz
^{15}N r.f. power	14.84 kHz
Ramp shape	80-100 % on ^{15}N
Duration	8.4 ms
90° ^{15}N flip pulses	
R.f. power	35.71 kHz
Duration	7 µs
Water suppression	
T delay	66 ms
Spoil pulse duration	1.2 ms
Spoil r.f. power	84.49 kHz
Spoil ramp shape	100-50 %
First pulse duration in train	71.4 ms
Second pulse duration in train	42 ms
Train r.f. power	29.13 kHz
Loops through train (*n*)	2
^{15}N-^{1}H CP step	
^{15}N r.f. power	29.77 kHz
^{1}H r.f. power	73.17 kHz
Ramp shape	100-80 % on ^{1}H
Duration	400 µs
Spin-echo step	
^{1}H pulse r.f. power	83.33 kHz
^{1}H pulse duration	6 µs
Acquisition	
WALTZ-16 r.f. power on ^{15}N	6.01 kHz
WALTZ-16 pulse duration	100 µs

Acquisition time	30 ms
Experimental time	
Experimental time	87.5 hours

Table S5 Set of pulse program parameters used for the acquisition of the 3D (H)CA(CO)NH spectrum of reprotonated pili at 900 MHz ^{1}H Larmor frequency and 40 kHz magic-angle spinning. The radio frequency (r.f.) power values were only determined for the high-power pulses via nutation experiments, the other r.f. powers are calculated assuming a linear amplifier and are therefore only estimates. The employed experiment with corresponding pulse sequence is described in detail in the literature[4,5].

Parameter	Reprotonated pili
Recycle delay	1 s
90° initial ^{1}H excitation pulse	
R.f. power	83.33 kHz
Duration	3 µs
^{1}H-^{13}CA CP step	
^{1}H r.f. power	73.17 kHz
^{13}CA r.f. power	27.23 kHz
Ramp shape	80-100 % on ^{1}H
Duration	6 ms
^{13}CA evolution time	
XiX r.f. power on ^{1}H	8.55 kHz
XiX pulse duration	200 µs
^{15}N inversion r.f. power	35.71 kHz
^{15}N inversion pulse duration	14 µs
Maximum acquisition time	5.76 ms
^{13}CA-^{13}CO DREAM CP step	
DREAM pulse r.f. power	12.37 kHz
DREAM pulse shape	Ramp 80-100 %
DREAM pulse duration	8.5 ms
Trim pulse r.f. power	54.32 kHz
Trim pulse duration	3.8 µs
^{13}CO-^{15}N CP step	
^{13}CO r.f. power	24.26 kHz
^{15}N r.f. power	17.51 kHz
Ramp shape	80-100 % on ^{15}N
Duration	8 ms
^{15}N evolution time	
WALTZ-16 r.f. power on ^{1}H	9.59 kHz
WALTZ-16 pulse duration	50 µs
180° XY r.f. power on ^{13}C	54.31 kHz
180° XY r.f. pulse duration	9.4 µs
180° XY delay	1 ms
Maximum acquisition time	12.6 ms
90° ^{15}N flip pulses	
R.f. power	35.71 kHz
Duration	7 µs
Water suppression	
T delay	77 ms
Spoil pulse duration	1.2 ms
Spoil r.f. power	85.47 kHz
Spoil ramp shape	100-50 %
First pulse duration in train	44 ms
Second pulse duration in train	66 ms
Train r.f. power	16 kHz
Loops through train (*n*)	2
^{15}N-^{1}H CP step	
^{15}N r.f. power	25.75 kHz
^{1}H r.f. power	73.59 kHz
Ramp shape	100-80 % on ^{1}H
Duration	400 µs
Spin-echo step	
^{1}H pulse r.f. power	83.33 kHz
^{1}H pulse duration	6 µs
Acquisition	
WALTZ-16 r.f. power on ^{15}N	5.7 kHz
WALTZ-16 pulse duration	100 µs
Acquisition time	33 ms
Experimental time	
Experimental time	56.5 hours

Table S6 Set of pulse program parameters used for the acquisition of the 3D (H)CO(CA)NH spectrum of reprotonated pili at 900 MHz ^{1}H Larmor frequency and 40 kHz magic-angle spinning. The radio frequency (r.f.) power values were only determined for the high-power pulses via nutation experiments, the other r.f. powers are calculated assuming a linear amplifier and are therefore only estimates. The employed experiment with corresponding pulse sequence is described in detail in the literature[4,5].

Parameter	Reprotonated pili
Recycle delay	
Recycle delay	1.16 s
90° initial ^{1}H excitation pulse	
R.f. power	83.33 kHz
Duration	3 µs
^{1}H-^{13}CO CP step	
^{1}H r.f. power	72.75 kHz
^{13}CO r.f. power	28.02 kHz
Ramp shape	80-100 % on ^{1}H
Duration	5.7 ms
^{13}CO evolution time	
XiX r.f. power on ^{1}H	8.45 kHz
XiX pulse duration	200 µs
^{15}N inversion r.f. power	35.71 kHz
^{15}N inversion pulse duration	14 µs
Selective ^{13}C r.f. power	9.38 kHz
Selective ^{13}C pulse duration	400 µs
Selective ^{13}C pulse shape	Gauss cascade Q3
Maximum acquisition time	10.5 ms
^{13}CO-^{13}CA DREAM CP step	
Trim pulse r.f. power	54.32 kHz
Trim pulse duration	3.8 µs
DREAM pulse r.f. power	12.16 kHz
DREAM pulse shape	Ramp 100-80 %
DREAM pulse duration	8.7 ms
^{13}CA-^{15}N CP step	
^{13}CA r.f. power	25.70 kHz
^{15}N r.f. power	14.62 kHz
Ramp shape	80-100 % on ^{15}N
Duration	8.4 ms
^{15}N evolution time	
WALTZ-16 r.f. power on ^{1}H	9.48 kHz
WALTZ-16 pulse duration	50 µs
180° XY r.f. power on ^{13}C	54.32 kHz
180° XY r.f. pulse duration	9.4 µs
180° XY delay	1 ms
Maximum acquisition time	12.1 ms
90° ^{15}N flip pulses	
R.f. power	35.71 kHz
Duration	7 µs
Water suppression	
T delay	77 ms
Spoil pulse duration	1.2 ms
Spoil r.f. power	84.49 kHz
Spoil ramp shape	100-50 %
First pulse duration in train	74.8 ms
Second pulse duration in train	44 ms
Train r.f. power	15.91 kHz
Loops through train (n)	2
^{15}N-^{1}H CP step	
^{15}N r.f. power	28.24 kHz
^{1}H r.f. power	75.74 kHz
Ramp shape	100-80 % on ^{1}H
Duration	400 µs
Spin-echo step	
^{1}H pulse r.f. power	83.33 kHz
^{1}H pulse duration	6 µs
Acquisition	
WALTZ-16 r.f. power on ^{15}N	5.70 kHz

WALTZ-16 pulse duration	100 µs
Acquisition time	33 ms
Experimental time	
Experimental time	84.5 hours

Table S7 Set of pulse program parameters used for the acquisition of the 2D ^{1}H-^{15}N correlation spectra of reprotonated pili and redeuterated pili at 700 MHz ^{1}H Larmor frequency and 40 kHz magic-angle spinning. The radio frequency (r.f.) power values were only determined for the high-power pulses via nutation experiments, the other r.f. powers are calculated assuming a linear amplifier and are therefore only estimates. The employed experiment with corresponding pulse sequence is described in detail in the literature[4,5].

Parameter	Reprotonated pili	Redeuterated pili
Recycle delay		
Recycle delay	1.2 s	1.2 s
90° initial ^{1}H excitation pulse		
R.f. power	83.33 kHz	83.33 kHz
Duration	3 µs	3 µs
^{1}H-^{15}N CP step		
^{1}H r.f. power	76.00 kHz	76.00 kHz
^{15}N r.f. power	29.37 kHz	28.96 kHz
Ramp shape	80-100 % on ^{1}H	80-100 % on ^{1}H
Duration	1 ms	0.9 ms
^{15}N evolution time		
WALTZ-16 r.f. power ^{1}H	8.33 kHz	7.17 kHz
WALTZ-16 pulse duration	50 µs	50 µs
180° XY r.f. power on ^{13}C	55.01 kHz	55.01 kHz
180° XY r.f. pulse duration	9.7 µs	10 µs
180° XY delay	0.5 ms	0.5 ms
Maximum acquisition time	42 ms	42 ms
90° ^{15}N flip pulses		
R.f. power	35.71 kHz	35.71 kHz
Duration	7 µs	7 µs
Water suppression		
T delay	66 ms	66 ms
Spoil pulse duration	1 ms	1 ms
Spoil r.f. power	83.33 kHz	71.75 kHz
Spoil ramp shape	50-100 %	50-100 %
First pulse duration in train	66 ms	33 ms
Second pulse duration in train	44 ms	22 ms
Train r.f. power	27.91 kHz	20.22 kHz
Loops through train (*n*)	2	2
^{15}N-^{1}H CP step		
^{15}N r.f. power	29.37 kHz	28.96 kHz
^{1}H r.f. power	76.00 kHz	76.00 kHz
Ramp shape	100-80 % on ^{1}H	100-80 % on ^{1}H
Duration	450 µs	450 µs
Spin-echo step		
^{1}H pulse r.f. power	83.33 kHz	83.33 kHz
^{1}H pulse duration	6 µs	6 µs
Acquisition		
WALTZ-16 r.f. power on ^{15}N	5.10 kHz	5.03 kHz
WALTZ-16 pulse duration	70 µs	70 µs
180° XY r.f. power on ^{13}C	55.01 kHz	55.01 kHz
180° XY r.f. pulse duration	9.7 µs	10 µs
180° XY delay	0.5 ms	0.5 ms
Acquisition time	40 ms	40 ms
Experimental time		
Experimental time	10.5 hours	11 hours

Table S8 Set of pulse program parameters used for the acquisition of the 3D (H)CANH spectra of reprotonated pili and redeuterated pili at 700 MHz ^{1}H Larmor frequency and 40 kHz magic-angle spinning. The radio frequency (r.f.) power values were only determined for the high-power pulses via nutation experiments, the other r.f. powers are calculated assuming a linear amplifier and are therefore only estimates. The employed experiment with corresponding pulse sequence is described in detail in the literature[4,5].

Parameter	Reprotonated pili	Redeuterated pili
Recycle delay		
Recycle delay	1.2 s	1.2 s
90° initial ^{1}H excitation pulse		
R.f. power	83.33 kHz	83.33 kHz
Duration	3 µs	3 µs
^{1}H-^{13}CA CP step		
^{1}H r.f. power	78.67 kHz	77.77 kHz
^{13}CA r.f. power	28.44 kHz	28.44 kHz
Ramp shape	80-100 % on ^{1}H	80-100 % on ^{1}H
Duration	6 ms	6.3 ms
^{13}CA evolution time		
WALTZ-16 r.f. power on ^{1}H	8.33 kHz	7.17 kHz
WALTZ-16 pulse duration	50 µs	50 µs
^{15}N inversion r.f. power	35.71 kHz	35.71 kHz
^{15}N inversion pulse duration	14 µs	14 µs
Maximum acquisition time	6 ms	6 ms
^{13}CA-^{15}N CP step		
^{13}CA r.f. power	23.93 kHz	23.93 kHz
^{15}N r.f. power	16.51 kHz	17.25 kHz
Ramp shape	80-100 % on ^{15}N	80-100 % on ^{15}N
Duration	9 ms	10.5 ms
^{15}N evolution time		
WALTZ-16 r.f. power on ^{1}H	8.33 kHz	7.17 kHz
WALTZ-16 pulse duration	50 µs	50 µs
180° XY r.f. power on ^{13}C	55.01 kHz	55.01 kHz
180° XY r.f. pulse duration	10 µs	10 µs
180° XY delay	0.5 ms	0.5 ms
Maximum acquisition time	13.4 ms	13.4 ms
90° ^{15}N flip pulses		
R.f. power	35.71 kHz	35.71 kHz
Duration	7 µs	7 µs
Water suppression		
T delay	44 ms	44 ms
Spoil pulse duration	1 ms	1 ms
Spoil r.f. power	83.33 kHz	71.75 kHz
Spoil ramp shape	100-50 %	100-50 %
First pulse duration in train	44 ms	33 ms
Second pulse duration in train	74.8 ms	56.1 ms
Train r.f. power	27.91 kHz	22.69 kHz
Loops through train (*n*)	2	2
^{15}N-^{1}H CP step		
^{15}N r.f. power	29.37 kHz	28.96 kHz
^{1}H r.f. power	76.88kHz	78.67 kHz
Ramp shape	100-80 % on ^{1}H	100-80 % on ^{1}H
Duration	450 µs	450 µs
Spin-echo step		
^{1}H pulse r.f. power	83.33 kHz	83.33 kHz
^{1}H pulse duration	6 µs	6 µs
Acquisition		
WALTZ-16 r.f. power on ^{15}N	5.10 kHz	5.03 kHz
WALTZ-16 pulse duration	70 µs	70 µs
Acquisition time	33 ms	33 ms
Experimental time		
Experimental time	20 hours	21 hours

Table S9 Chemical shifts of the reprotonated pilus sample. The main set of chemical shifts and additional peak splittings are listed. The chemical shifts were acquired at 900 MHz ^{1}H Larmor frequency, 40 kHz magic-angle spinning, and an internal sample temperature of 22±1 °C. Chemical shifts are deposited in the Biological Magnetic Resonance Bank (BMRB entry 27861).

		Polymorph A				Main set				Polymorph B			
		H^N	N	C	Cα	H^N	N	C	Cα	H^N	N	C	Cα
1	Ala					-	-	-	-				
2	Ala					-	-	-	-				
3	Thr					-	-	173.42	62.16			175.26	61.69
4	Thr					8.7	122.97	174.62	61.47	8.81	116.18	173.17	60.77
5	Val					9.45	120.81	174.45	58.36	8.4	114.56	174.74	59.04
6	Asn			177.05		8.66	119.89	172.46	55.14	8.33	123.97		54.56
7	Gly	6.72	114.47	170.76	46.81	7.47	111.43	171.25	45.66				
8	Gly	6.44	104.03	171.47	46.33	5.86	106.94	172.44	46.47			172.5	46.25
9	Thr	8.42	117.67	172.49	62	8.78	119.84	172.27	62.04	8.67	118.99	172.28	61.73
10	Val					9.14	124.12	172.11	60.21	9.06	124.14	172.26	60.13
11	His					8.34	123.66	173.16	53.18	8.63	121.4	173.19	53.22
12	Phe					9.61	123.29	176.01	57.26				
13	Lys					8.78	122.94	174.35	53.33				
14	Gly					7.94	108.92	173.31	46.51				
15	Glu	7.77	115.09	173.83	55.87	7.72	117.79	173.26	56.55				
16	Val	8.79	122.88	176.85	59.97	8.99	125.73	176.91	60.07				
17	Val			174.43	58.71	9.31	121.12	174.24	58.76				
18	Asn	9.37	123.82	173.05	51.2	9.24	122.69	173.55	51.12				
19	Ala	7.5	120.28	176.72	50.9	7.55	121.27	176.86	50.9				
20	Ala					7.17	118.33	174.53	54.48				
21	Cys					6.07	104.02	171.35	53.22			171.5	53.06
22	Ala					9.06	123.3	175.69	49.94	9.25	123.96	175.6	50.12
23	Val					9.09	126.07	177	65.32				
24	Asp					8.47	127.96	177.23	56.15			177.33	56.37
25	Ala					9.56	127.51	178.77	56.37	9.47	127.48		
26	Gly					8.71	104.52	175.1	46.21				
27	Ser					8.16	113.24	174.4	59.12			174.65	59.11
28	Val					7.25	116.39	176.33	64.53	7.22	115.64		64.52
29	Asp					8.09	123.68	174.9	54.6				
30	Gln					7.84	120.35	174	54.8				
31	Thr					8.96	119.19	173.44	62.08				
32	Val					9.34	131.54	173.78	61.1				
33	Gln					8.81	128.51	174.88	54.15				
34	Leu					9.93	127.54	177.11	57.55				
35	Gly					8.64	104.71	172.19	43.79	8.74	104.97		
36	Gln			175.2	53.76	6.74	111.29	176.11	53.79				
37	Val	8.52	118.54	172.41	59.84	8.41	117.48	172.46	59.86				
38	Arg	8.69	126.86	179.46	55.67	8.77	127.08	179.55	55.71			179.58	55.76
39	Thr					9.05	115.51	178.56	65.53	9.18	115.51	178.43	65.53
40	Ala					8.3	123.21	179.06	54.49				
41	Ser					7.56	112.31	172.79	59.4				
42	Leu					7.36	123.5	174.6	51.74				
43	Ala					7.68	118.74	176.71	53.26				
44	Gln					7.05	112.07	172.74	53.36				
45	Glu					8.53	120.27	177.29	57.04				
46	Gly					8.65	115.15	173.76	44.43				
47	Ala					7.68	123.87	175.62	52.74				
48	Thr					8.11	108.15	175.33	58.32				
49	Ser					8.82	117.76	174.63	57.65				
50	Ser					8.18	114.43	172.71	59.67				
51	Ala					8.69	124.11	176.8	51.15				

52	Val					8.59	114.59	175.25	60.95				
53	Gly					9.07	111.92	173.66	44.71				
54	Phe					8.42	117.22	171.12	57.24				
55	Asn					9.24	117.33	175.25	51.54				
56	Ile					8.8	119.89	173.74	61.09				
57	Gln					8.83	128.56	173.6	55.11	8.99	128.49	173.99	55.33
58	Leu					9.2	127.48	173.76	52.86				
59	Asn					9.51	118.79	176.01	50.7			176.47	50.66
60	Asp					9.63	118.01	174.52	54.16	9.49	117.39	174.73	54.14
61	Cys					8.65	112.2	177.56	56.32				
62	Asp					8.12	122.26	178.05	52.27				
63	Thr					8.68	116.24	176.43	63.07				
64	Asn					8.98	119.78	176.27	54.71				
65	Val					7.87	120.67	175.49	63.98				
66	Ala					7.39	119.49	173.69	52.09				
67	Ser					9.01	111.94	174.87	58.48	9.06	112.32		
68	Lys					8.74	122.78	174.37	55.09				
69	Ala					8.42	119.36	176.2	49.77				
70	Ala					8.82	123.65	174.46	51.45				
71	Val			174.02	61.14	8.95	119.89	174.03	60.87				
72	Ala	8.88	129.5	176.98	49.82	8.08	127.77	176.2	49.89				
73	Phe	10.02	123.15	174.44	57.11	9.95	120.88	174.43	56.98				
74	Leu	9.29	124.03		53.09	9.27	123.48	174.87	53.15				
75	Gly					8.1	111.86	169.7	44.43				
76	Thr					8.66	117.57	173.23	62.92				
77	Ala					8.39	130.47	177.68	50.44				
78	Ile					8.23	118.26	174.81	64.6				
79	Asp					7.3	112.88	176.11	54.18				
80	Ala					8.31	120.23	178.5	54.24				
81	Gly					8.78	105.66	174.06	44.82				
82	His					7.92	122.16	174.6	54.99				
83	Thr					8.14	109.29	174.59	63.43				
84	Asn					7.62	116.88	174.13	51.24				
85	Val					7.33	121.28	172.97	61.95				
86	Leu					9.91	131.74	175.84	54.74				
87	Ala					9.25	126.64	175.82	51.14				
88	Leu					8.62	117.82	179.38	53.55				
89	Gln					8.92	114.37	176.63	54.43				
90	Ser					8.61	112.54	173.14	63.47				
91	Ser					7.71	115.59	173.3	58.27				
92	Ala					8.86	120.26	175.09	54.24				
93	Ala					7.15	115.55	179.34	50.43				
94	Gly					9.36	107.22	174.9	46.25				
95	Ser					7.85	115.61	174.33	58.91				
96	Ala					8.89	126.99	177	52.94				
97	Thr					7.25	110.39	173.18	60.34				
98	Asn					9.03	113.09	173.64	53.86				
99	Val					7.26	112.75	172.83	59.95				
100	Gly					8.57	111.84	172.35	44.9				
101	Val					8.64	121.21	174.97	61.45				
102	Gln					9.26	123.45	173.46	54.13				
103	Ile					9.46	123.27	173.91	60.48				
104	Leu					9.47	126.24	177.98	52.22				
105	Asp					8.46	118.21	177.46	51.47				
106	Arg					7.91	110.43	177.61	57.84				
107	Thr					8.93	117.79	173.55	61.77				
108	Gly					8.63	109.78	173.46	45.55				
109	Ala					7.26	123.28	176.19	51.09				
110	Ala					8.25	124.04	178.6	51.4				

111	Leu					8.81	126.52	177.14	54.53				
112	Thr					8.04	118.4	174.68	64.47	8.34	118.39	174.8	64.42
113	Leu					7.53	130.88	177.67	54.72	7.52	130.05	177.77	54.87
114	Asp					7.82	117.46	177.43	53.21	8.61	117.87	176.82	51.09
115	Gly					8.84	109.68	172	46.71				
116	Ala					7.99	119.59	175.47	51.27				51.04
117	Thr					8.13	120.4	174.03	62	7.98	120.44	173.87	61.96
118	Phe					8.79	126.58	176.9	60.03	8.79	125.97	176.82	59.96
119	Ser					9.4	115.73	172.65	58.12				
120	Ser					8.89	114.12	176.43	60.42				
121	Glu					9.18	128.22	176.8	57	9.18	128.03		56.7
122	Thr					9.05	119.6	172.4	62.26				
123	Thr					9.31	124.31	173.64	64.1				
124	Leu					8.42	126.33	178.04	53.74				
125	Asn					9.38	116.14	175.42	51.59				
126	Asn					9.66	121.5	178.04	54.24				
127	Gly	9.29	113.28	174.75	45.77	9.61	114.59	174.51	46.47				
128	Thr	9.21	125.17	175.62	62.96	8.88	125.68	174.35	63.64				
129	Asn	10.21	126.58	172.32	52.82	9.93	124.28	172.93	52.35				
130	Thr					9.19	120.05	172.24	62.17				
131	Ile					8.79	126.68	173.87	57.04				
132	Pro					-	124	174.62	62.53				
133	Phe					8.19	115.43	176.14	56.71				
134	Gln					8.86	117.37	174.57	54.36				
135	Ala					9.35	121.31	175.27	49.84				
136	Arg					8.36	114.27	172.99	53.63				
137	Tyr					9.17	118.73	174.94	58.13				
138	Phe					9.08	123.05	173.38	56.29				
139	Ala					8.62	130.82	175.74	50.5				
140	Thr					8.24	112.53	173.98	60.91				
141	Gly					7.49	110.58	169.6	44.15				
142	Ala					8.33	122.04	177.26	52.15				
143	Ala					8.5	129.9	177.97	52.48				
144	Thr					7.78	110.79	171.36	58.26				
145	Pro					-	128.2	176.41	62.65				
146	Gly					8.69	107.5	172.5	44.33	8.88	107.63		44.4
147	Ala					8.7	123.13	177.09	54.07				53.92
148	Ala					8.76	128.63	173.83	51.13	8.65	128.44		
149	Asn					8.49	121.08	173.81	51.27				
150	Ala					8.95	121.27	175.48	52.21				
151	Asp					9.42	118.74	172.98	54.27				
152	Ala					9.04	122.14	176.1	51.19				
153	Thr	8.82	110.77	173.34	60.3	9	111.18	173.87	60.43				
154	Phe	8.46	114.94	174.37	54.96	8.59	115.21	173.69	55.46				
155	Lys					8.64	119.57	174.3	54.1	8.53	120.5		53.21
156	Val					8.87	120.09	173.06	60.94	8.87	125.45	173.38	60.18
157	Gln					9.16	126.35	173.84	53.48	9.04	123.8	173.71	53.43
158	Tyr					9.16	126.26	175.69	56.52			175.55	55.74
159	Gln					8.6	125.85	179.27	57.59	8.67	126.81		57.28

References

1. Puorger, C., Vetsch, M., Wider, G. & Glockshuber, R. Structure, folding and stability of FimA, the main structural subunit of type 1 pili from uropathogenic *Escherichia coli* strains. *J Mol Biol* **412**, 520-535 (2011).

2. Hospenthal, M.K. et al. The cryoelectron microscopy structure of the type 1 chaperone-usher pilus rod. *Structure* **25**, 1829-1838 (2017).

3. Kabsch, W. & Sander, C. Dictionary of protein secondary structure: Pattern-recognition of hydrogen-bonded and geometrical features. *Biopolymers* **22**, 2577-2637 (1983).

4. Chevelkov, V. et al. Proton-detected MAS NMR experiments based on dipolar transfers for backbone assignment of highly deuterated proteins. *J Magn Reson* **242**, 180-188 (2014).

5. Fricke, P. et al. Backbone assignment of perdeuterated proteins by solid-state NMR using proton detection and ultrafast magic-angle spinning. *Nat Protoc* **12**, 764-782 (2017).
